# NIR-IIb fluorescence antiangiogenesis copper nano-reaper for enhanced synergistic cancer therapy

**DOI:** 10.1186/s12951-024-02343-5

**Published:** 2024-02-19

**Authors:** Wenling Li, Huan Xin, Wenjuan Gao, Pengjun Yuan, Feixue Ni, Jingyi Ma, Jingrui Sun, Jianmin Xiao, Geng Tian, Lu Liu, Guilong Zhang

**Affiliations:** School of Pharmacy, Shandong Technology Innovation Center of Molecular Targeting and Intelligent Diagnosis and Treatment, Binzhou Medical University, Yantai, 264003 P.R. China

**Keywords:** Copper nano-reaper, Photothermal therapy, Anti-angiogenesis, Antitumor immunity, Tumor metastasis

## Abstract

The formation of blood vessel system under a relatively higher Cu^2+^ ion level is an indispensable precondition for tumor proliferation and migration, which was assisted in forming the tumor immune microenvironment. Herein, a copper ions nano-reaper (LMDFP) is rationally designed not only for chelating copper ions in tumors, but also for combination with photothermal therapy (PTT) to improve antitumor efficiency. Under 808 nm laser irradiation, the fabricated nano-reaper converts light energy into thermal energy to kill tumor cells and promotes the release of D-penicillamine (DPA) in LMDFP. Photothermal properties of LMDFP can cause tumor ablation in situ, which further induces immunogenic cell death (ICD) to promote systematic antitumor immunity. The released DPA exerts an anti-angiogenesis effect on the tumor through chelating copper ions, and inhibits the expression of programmed death ligand 1 (PD-L1), which synergizes with PTT to enhance antitumor immunity and inhibit tumor metastasis. Meanwhile, the nanoplatform can emit near-infrared-IIb (NIR-IIb) fluorescence under 980 nm excitation, which can be used to track the nano-reaper and determine the optimal time point for PTT. Thus, the fabricated nano-reaper shows powerful potential in inhibiting tumor growth and metastasis, and holds great promise for the application of copper nanochelator in precise tumor treatment.

**Supplementary Information:**

The online version contains supplementary material available at 10.1186/s12951-024-02343-5.

## Introduction

The progressive growth of the tumor leads to insufficient nutrition for tumor cells from normal blood vessel system. It is urgent for growing tumor to generate a new blood vessel system that is necessary for tumor metastasis [1, 2]. Toward this end, blocking the generation of tumor blood vessels for starvation treatment has received tremendous attention in recent years [3–5]. Copper plays a critical role in tumor progression [6, 7], which is not only in the secretion of the angiogenic factor but also in the proliferation and migration of tumor cells [8–10]. Recently, the strategy of chelating copper ions for antivascular therapy has been continuously reported [11, 12]. Besides, Vittorio group found that chelating Cu^2+^ can inhibit the expression of PD-L1 to enhance the antitumor immune response [13]. D-penicillamine (DPA) is a copper ionic chelator commonly used in clinical practice and is highly effective and capable. As a small molecular chelating agent, DPA is easy to load and release and has been widely studied. However, nonspecific release without tumor targeting increases the systemic toxicity of the body and causes various side effects. In addition, the penetration of tumor cells to chelate intracellular Cu^2+^ is still facing great challenges, which further limits their application in antitumor therapy [14–17].

Photothermal therapy (PTT) as an effective non-invasive tumor therapy strategy can effectively ablate tumors while avoiding damage to normal tissue for tumor cells are more sensitive to temperature change [18–20]. Commonly used PTT materials, including precious metal nanomaterials, carbon-based nanomaterials, semiconductor nanomaterials, and lanthanide-doped nanocrystals (LDNP), of which lanthanide-doped nanocrystals can also demonstrate excellent NIR-IIb fluorescent properties [21, 22]. NIR-IIb fluorescence imaging provides high quality photon attenuation, tissue autofluorescence, and significantly reduced scattering, which opens many exciting new imaging avenues for drug delivery and cancer detection. Hence, the use of lanthanide-doped agents is of great significance for photothermal and NIR-IIb fluorescence applications in vivo. Most of cancer-related deaths are due to metastasis [23, 24]. In recent years, cancer immunotherapy by stimulating the patient’s innate immune system has shown great prospects in inhibiting metastasis [25, 26]. It has been reported that photothermal tumor ablation can promote antitumor immune response and induce immunogenic cell death (ICD) by delivering tumor associated antigen (TAA) [27, 28]. In this process, photothermal action induces tumor cell death and causes the release of TAA by tumor cells. Then, dendritic cells (DCs) capture these antigens that migrate to the spleen or lymph nodes, presenting antigens to T cell receptors by major histocompatibility complex (MHC) antigen compounds, initiating T cell-mediated cancer immune [29, 30]. However, considering the heat endurance of the single photothermal treatment, it might not be realistic to eliminate all tumor cells that usually cause metastasis through the tumor vasculature network [31–34]. Therefore, it is urgent to design a single nanoplatform that can induce ICD, promote antitumor immune response through photothermal ablation, and disrupt the tumor vasculature network to inhibit tumor metastasis.

**Scheme 1:**
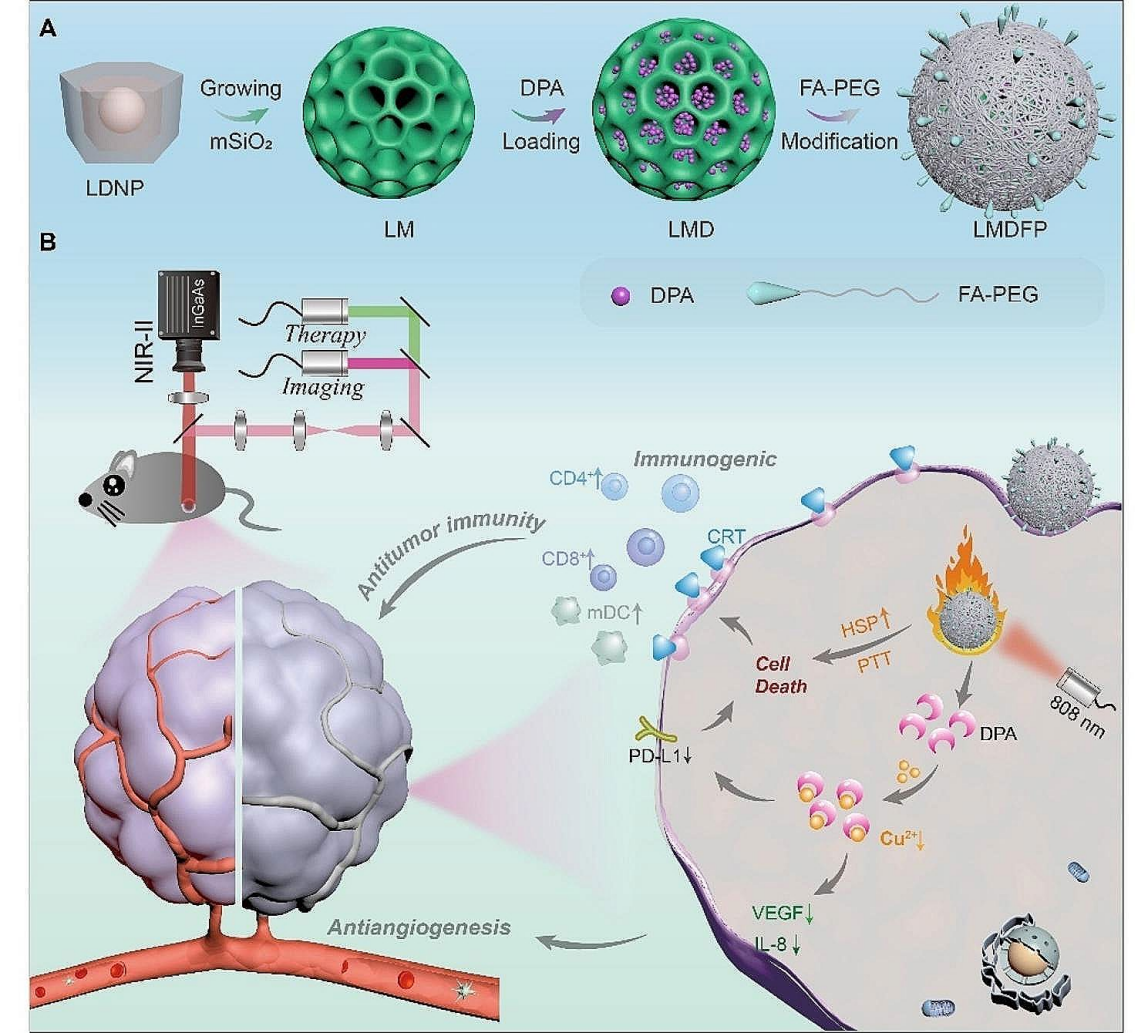
(**A**) Fabrication of the LMDFP nanodrug. (**B**) Schematic illustration of the NIR-IIb fluorescence antiangiogenesis copper nano-reaper for enhanced synergistic cancer therapy

Herein, we designed the LDNP@mSiO_2_-DPA@FA-PEG (LMDFP) nano-reaper with photothermal and antiangiogenic effects to enhance antitumor ability and restrain metastasis. LDNP consisted of NaYF_4_:Yb,Er@NaGdF_4_@NaNdF_4_ has independent NIR-IIb downshifting fluorescence and photothermal conversion effect. Outer-shell NaNdF_4_ dominates photothermal conversion that converts 808 nm light energy into heat for photothermal therapy. The NaYF_4_:Yb,Er inner-core has been employed for NIR-IIb fluorescence imaging to monitor drug delivery and determine the optimal triggering time of PTT. Combined with the superiority of mesoporous silica (mSiO_2_), LDNP@mSiO_2_ (LM) can also be used for loading the copper ion chelating agent DPA (LMD). By further wrapping FA-PEG on the surface of LMD, LMDFP nanodrugs with tumor targeting were successfully obtained (Scheme 1A). Under the 808 nm laser irradiation, the increased temperature of the nanodrug triggered the release of DPA further for antitumor through PTT and anti-angiogenesis effect. Meanwhile, the exposure to TAA caused by PTT and the down-regulation of PD-L1 expression induced by DPA jointly enhance the immune system to suppress distant tumor metastasis (Scheme 1B).

## Results and discussion

**Fig. 1:**
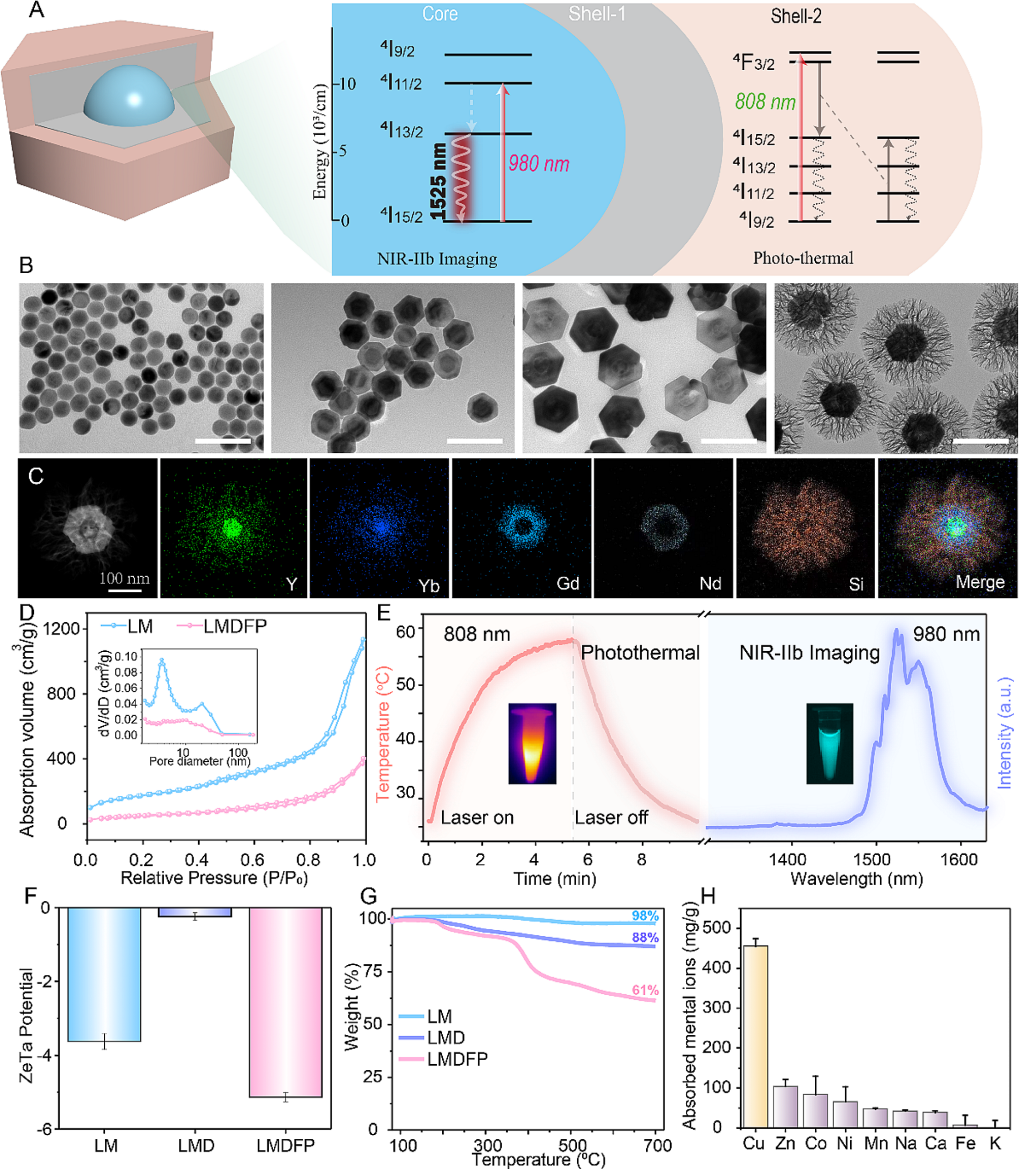
(**A**) Structure of the LDNP and the proposed energy transfer mechanisms in the multi-layer core/shell nanocrystal. (**B**) Transmission electron microscopy (TEM) images of NaYF_4_:Yb,Er, NaYF_4_:Yb,Er@NaGdF_4_, NaYF_4_:Yb,Er@NaGdF_4_@NaNdF_4_, NaYF_4_:Yb,Er@NaGdF_4_@NaNdF_4_@mSiO_2_. (Scale bar: 200 nm). (**C**) HAADF-STEM image with corresponding EDS elemental mapping images. (**D**) The N_2_ sorption isotherms and the corresponding pore size distribution curve before and after coating FA-PEG. (**E**) Photothermal/downshifting emission spectra of LDNPs under 808 and 980 nm excitation, respectively. (**F**) Zeta potential of LM, LMD, and LMDFP. (**G**) Thermogravimetric analysis of LM, LMD and LMDFP. (**H**) The chelating capacity of DPA to different metal ions

As shown in Fig. 1A, NaYF_4_:Yb,Er@NaGdF_4_@NaNdF_4_ core-shell-shell nanocrystal is designed with independent orthogonal downshifting and photothermal conversion effect. Transmission electron microscopy (TEM) showed high monodispersity of the NaYF_4_:Yb,Er core nanoparticles with a uniform size of ∼65 nm (Fig. 1B). After epitaxial growth with various functional shells by the successive layer-by-layer strategy [35], the core-multishell nanoparticles (∼150 nm) were obtained. Afterward, the ordered mesoporous SiO_2_ shell with a thickness of ∼55 nm was uniformly coated around the core-multishell LDNP. HAADF-STEM image and corresponding EDS elemental mapping images show the composition of the LM nanostructures (Fig. 1C). The N_2_ adsorption-desorption isotherm curve indicates that LM and LMDFP has a uniform mesopore size of ∼3.5 nm and ∼11.0 nm (Fig. 1D). Meanwhile, the Brunauer-Emmett-Teller (BET) surface area of the LM and LMDFP is calculated to be 624.5 m^2^/g and 190.6 m^2^/g, respectively. The abundance of mesopores also disappeared for LMDFP, which were assigned to the successful loading of DPA and the surface modification of FA-PEG. Fourier transform infrared (FT-IR) spectra of FA-PEG-coated LMDFP nanoparticles (Additional file 1: Fig. S1) convey the presence of characteristic absorption peaks of FA-PEG, which can be considered direct evidence of polymer coating. The 1530 nm downshifting emissions from the NaYF_4_:Yb,Er core can be realized with an excitation of 980 nm, which can be used for NIR-IIb fluorescence imaging (Fig. 1E). Nd^3+^ ions have abundant and close energy levels that boost the multiphonon decay processes accompanied by efficient heat generation. Therefore, the NaNdF_4_ outer-shell with strong 808 nm light absorption was designed as the photothermal agent. Owing to the presence of the inert NaGdF_4_ energy barrier layer, the outer NaNdF_4_ shell has little influence on NaYF_4_:Yb,Er luminescence (Additional file 1: Fig. S2). Furthermore, to detect DPA loading and FA-PEG decoration, ζ-potential, hydrodynamic radii and thermogravimetric analysis were used. The ζ-potential and nanoparticle size variations among LM, LMD, and LMDFP indicated successful synthesis at each step (Fig. 1F and Additional file 1: Fig. S3). Thermogravimetric analysis of LM, LMD, and LMDFP demonstrated the thermo-decomposition process of DPA and FA-PEG molecules, respectively (Fig. 1G).

The photothermal (PT) property of LMDFP was systematically investigated. Under various pumping powers of 808 nm irradiation, LMDFP at 200 µg/mL exhibits a power density-dependent heating behavior (Additional file 1: Fig. S4). By contrast, there is little increase in the temperature of deionized water, even under higher pumping powers of 4 W/cm^2^. In addition, the temperature change of LMDFP with various concentrations is examined under 808 nm laser irradiation, which further indicates that the LMDFP has an outstanding photothermal effect. It should be noted that the temperature of the LMDFP solution at 200 µg/mL reached more than 48 °C under laser irradiation for 800 s (Additional file 1: Fig. S4A). Furthermore, the temperature elevation and natural cooling curves of LMDFP are measured to examine the photothermal stability. As shown in Additional file 1: Fig. S4C, no obvious temperature decay is detected in five irradiation/cooling cycles, indicating that the LMDFP possessed outstanding photothermal stability. The photothermal conversion efficiency of LMDFP reached up to 31.6% upon 808 nm laser irradiation (Additional file 1: Fig. S5). Subsequently, to observe copper depletion in the complex physiological condition, the selectivity of the DPA chelator for capturing copper ions was studied in the presence of various biologically relevant metal ions solution. Figure 1H shows the high selectivity of DPA chelator toward copper over other metal ions. The superior copper capturing capacity of DPA can be attributed to the soft thiolate and amine donors for binding Cu^2+^ [36]. To demonstrate the controlled release of DPA, we studied the absorbance of DPA-Cu chelate upon 808 nm laser irradiation of LMDFP. As shown in Additional file 1: Fig. S6, the increased absorbance of DPA-Cu at 525 nm was observed under 808 nm laser irradiation at different times. These findings illustrate that upon irradiation with an 808 nm laser, photothermal effects effectively induced D-penicillamine continued release, which subsequently chelated with copper ions. Furthermore, the effect of nanodrugs concentration on the copper depletion ability of LMDFP was studied in the presence of copper ions solution. From Additional file 1: Fig. S7, it is observed that the consumption of copper gradually increases as the concentration of LMDFP is increased. These results confirmed that DPA had a high selectivity performance to chelate copper ions, which laid the foundation for LMDFP to selectively eliminate copper content in tumor.

**Fig. 2:**
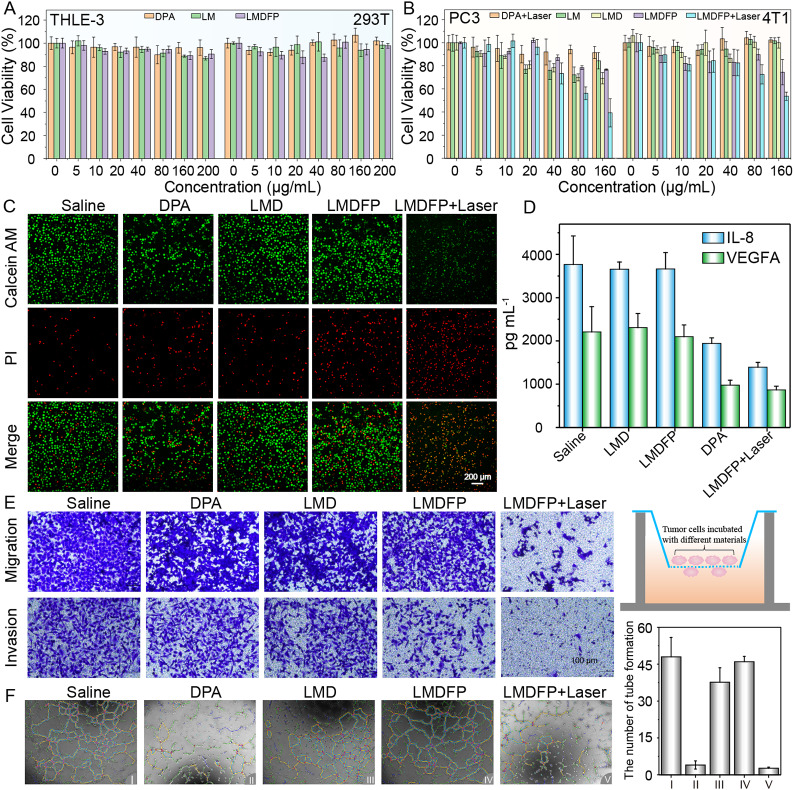
(**A, B**) Relative cell viability of THLE-3, 293T, PC-3, and 4T1 cells after incubation with different treatments at various concentrations for 24 h. (**C**) Confocal fluorescence microscopy (CLSM) observations: live (green) and dead (red) PC-3 cells stained by calcein-AM and PI after various treatments for 24 h. (**D**) Cytokine expression levels of proangiogenic mediators including IL-8 and VEGFA in PC-3 cells detected by ELISA. (**E**) PC-3 migration and invasion ability were tested using transwell assay after various treatments for 48 h. (**F**) Tube-formation abilities of C166 after different treatments for 48 h

The antitumor activity of LMDFP nano-reaper was initially investigated at a cellular level using the cell-counting kit-8 (CCK-8) assay [37]. To demonstrate cell biocompatibility, the THLE-3 (human liver epithelial cells) and 293T (human renal epithelial cells) cells were treated with different drugs at different doses (Fig. 2A and Additional file 1: Fig. S8). The cell survival rate was above 80% even after 48 h of co-incubation with LMDFP at a high concentration of 200 µg/mL, confirming the excellent biocompatibility of LMDFP. To study copper content and sensibility to DPA in different cancer cells, we selected four common cancer cell lines MCF-7 (human breast cancer cells), 4T1 (mouse breast cancer cells), HeLa (human cervical carcinoma cells) and PC-3 (human prostatic cancer cells). As illustrated in Additional file 1: Fig. S9A, the PC-3 cells and 4T1 cells are from opposite ends of the copper content range among the four types of cancer cell. Interestingly, cell viability results inidcated that both PC-3 and 4T1 cells have high sensitivity to the DPA, and they were concentration-dependent on DPA (Additional file 1: Fig. S9B). The results indicate that the sensitivity to DPA of these cell lines may have no direct link with cellular copper content, which does not mean that the higher the copper content in tumor cells, the more sensitive they are to copper ion chelator. Considering the high sensitivity to DPA, 4T1 and PC-3 cells were used in the following study in vivo and in vitro. Under 808 nm laser irradiation (1.5 W/cm^2^ for 5 min), the PC-3 and 4T1 cell viabilities significantly decreased and were highly dependent on the concentration of LMDFP nanodrugs (Fig. 2B and Additional file 1: Fig. S8). The inhibition effect of various treatment on tumor cells was further verified on PC-3 cells through the live/dead staining assay using propidium iodide and calcein-AM double-staining under confocal laser scanning microscope (CLSM) (Fig. 2C). The results revealed that the highest ratio of apoptotic cells was observed and the largest number of dead cells was detected in the LMDFP + Laser group. The copper depletion ability of LMDFP was also studied in the 4T1 cancer cells, it can be observed that the content of copper gradually decreases as the concentration of LMDFP is increased (Additional file 1: Fig. S10a). In order to further validate the ability of LMDFP nano-reaper to deplete copper in vivo, the clearance rate of copper ions was investigated in mice bearing 4T1 tumors. The LMDFP + Laser-treated group exhibited lower copper ion concentration compared to the saline group, indicating the excellent copper ion chelating action of our nano-reaper (Additional file 1: Fig. S10b). These findings illustrate that the cytotoxicity of LMDFP nano-reaper may be attributed to the synergistic action of effective PTT and copper elimination induced by DPA. Simultaneously, we used the nuclear fluorescent probe hoechst33342 and the fluorescence of fluorescein isothiocyanate (FITC) for verifying the location of the nucleus and nano-reapers. The results showed that the internalization of LMD-FITC and LMDFP-FITC is concentration-dependent, and the strongest green fluorescence intensity was observed in the cytoplasm of PC-3 cells treated with LMDFP-FITC nanodrugs, indicating excellent specific targeting ability in this group (Additional file 1: Fig. S11).

To further evaluate the inhibition of tumor migration and invasion induced by the copper nano-reaper, a transwell system in which 4T1 and PC-3 cells were treated with drugs was established. As illustrated in Fig. 2E and Additional file 1: Fig. S12, the migration and invasion ability in 4T1 and PC-3 cells were markedly decreased after LMDFP + Laser treatment, which was followed by the DPA group. Furthermore, the cell scratch assay was used to assess the cancer cell migration ability. Consistent with the transwell experiment, the cell scratch experiment manifested that the migration ability of PC-3 and 4T1 cells was observably inhibited after LMDFP + Laser treatment (Additional file 1: Fig. S13). The above results indicated that the PT effect generating from LDNPs under 808 nm irradiation and copper deficiency resulting from the chelation of DPA can substantially influence the motility of cancer cells. In addition, we evaluated the influence of different concentrations of DPA on the tube formation ability of C166 (mouse vascular endothelial cells) in Matrigel-coated plates. As shown in Additional file 1: Fig. S14, DPA obviously impaired the C166 tube formation even at the concentration of 50 µg/mL, and the tube formation ability is highly dependent on the concentration of DPA. Meanwhile, we further evaluated the influence of various materials on the tube formation ability of C166. In the control group and treatment groups of LMD and LMDFP, C166 formed evident tube-like networks in plates (Fig. 2F). In comparison, the tube-forming ability was severely inhibited in the DPA group and LMDFP + Laser group, in which most tubes remained open and an increased number of dissociative cells in plates. These results suggested that the DPA combinate PT effect enhanced the antiangiogenic efficacy in vitro. To observe the effect on tube formation caused by cancer cells after incubation with DPA, we collected the supernatant of the DPA-treated 4T1 and PC-3 cells culture medium to incubate C166 cells. As shown in Additional file 1: Fig. S15, tube formation was severely inhibited in the DPA-treated cancer group, indicating that DPA may affect the secretion of angiogenesis-related cytokines by reducing copper ions in cancer cells. VEGFA and IL-8 as proangiogenic factors play an essential role in the vascular angiogenesis of tumor [38]. Thus, we further assessed the expressions of these signaling factors in PC-3 cells to discuss possible antiangiogenetic mechanisms of nanodrugs. As expected, the VEGFA and IL-8 expressions detected by the ELISA kits were greatly reduced in PC-3 cells after treatment of DPA or LMDFP for 24 h (Fig. 2D), which confirmed DPA can inhibit the secretion of vascular angiogenesis cytokines. Meanwhile, we investigated the role of VEGFA protein in tumor vascular angiogenesis using western blotting assay (WB). As shown in Additional file 1: Fig. S16, the expression of VEGFA significantly decreased after treatment with various concentrations of LMDFP nano-reaper. Moreover, the inhibition of PD-L1 induced by the copper nano-reaper was also investigated at a cellular level. As is shown in Additional file 1: Fig. S17, after different treatment, the expression of PD-L1 decreased obviously in LMDFP + Laser group, reflecting the downregulation of PD-L1 through chelating copper ions caused by DPA released from LMDFP NPs after irradiation, indicating that LMDFP could inhibit tumor immune escape.

**Fig. 3:**
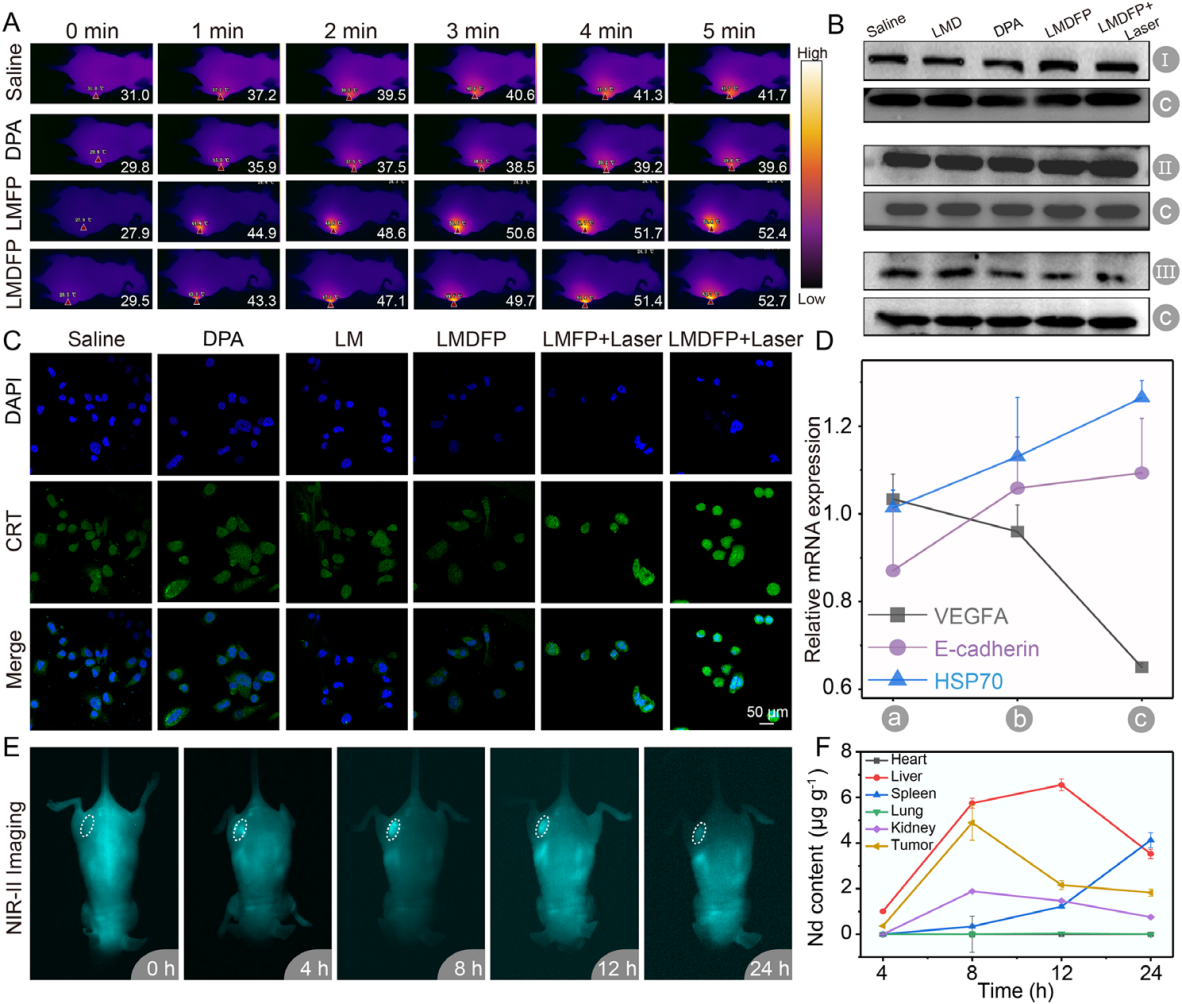
(**A**) Thermal image of PC-3 tumor loaded mice injected with different samples under 808 nm laser irradiation (1.5 W/cm^2^). (**B**) The protein expressions of VEGFA, E-cadherin and HSP70 in 4T1 cells analyzed by western blotting (I: E-cadherin, II: HSP70, III: VEGFA, C: GAPDH). (**C**) Fluorescence stain images of CRT expression in PC-3 cells with different treatments. (**D**) mRNA expressions of VEGFA, E-cadherin and HSP70 in PC-3 cells were tested by quantitative real-time PCR (a: saline, b: LMDFP, c: LMDFP + Laser). (**E**) In vivo NIR-IIb fluorescence imaging at different time points of the nude mice bearing PC-3 tumor after intravenous (i.v.) injection of LMDFP. (**F**) ICP-OES of the Nd element concentration in the major organs and tumors at different time points

The Photothermal ability of LMDFP was further observed under 808 nm laser irradiation (1.5 W/cm^2^) in vivo. Four tumor-bearing mice were administered with Saline, DPA, LMFP and LMDFP, respectively, followed by irradiation of 808 nm laser. A thermal imager was used to record the temperature changes of the tumor sites in real time. After 5 min irradiation, the maximal temperature of the tumor of the four mice injected with Saline, DPA, LMFP, and LMDFP increased to 41.7, 39.6, 52.4 and 52.7 °C, respectively (Fig. 3A). These results confirmed that the photothermal ability of LMDFP comes from LDNP, rather than mSiO_2_ and DPA. To further investigate the mechanism of vascular formation, tumor motility and tumor cell death caused by LMDFP + Laser, the characteristic proteins including VEGFA, E-cadherin and heat-shock protein 70 (HSP70) were investigated using western blotting (WB) assay. As is shown in Fig. 3B, after LMDFP + Laser treatment, the expression of VEGFA, a vital angiogenesis associated protein, decreased obviously. The expression of E-cadherin has an obvious relationship with contact inhibition, which is associated with increased cell motility and advanced stages of cancer [39]. Notably, the expression of E-cadherin in LMDFP + Laser group was significantly up-regulated compared to the saline and LMDFP groups. Meanwhile, the PT effect further enhanced the expression of HSP70, implying heat-shock-mediated cell death. Moreover, the expression of VEGFA, E-cadherin and HSP70 in mRNA level was also investigated through qRT-PCR. The results are shown in Fig. 3D, which was consistent with WB results. Together, these results confirmed that the LMDFP + Laser group prevents vascular formation, inhibits tumor motility, and induces tumor cell death in protein and mRNA levels. As one of the distinct biomarkers of the damage-associated molecular patterns (DAMPS), calreticulin (CRT) is detected to test whether LMDFP + Laser was capable of inducing ICD of cancer cells [40]. We determined CRT expression during ICD after incubation PC-3 or 4T1 cells with various nanodrugs. As imaged by CLSM (Fig. 3C and Additional file 1: Fig. S18), the level of CRT significantly upregulated in both LMFP + Laser and LMDFP + Laser groups. Furthermore, LMDFP + Laser group induced slightly stronger CRT expression compared to LMFP + Laser and LMDFP groups. Together, these results indicated that PT effect could indeed induce ICD and that copper ions chelator may also participate in tumor ICD.

NIR-II fluorescence imaging has attracted great attention in the real-time monitoring of drug delivery [41–43]. Hence, we developed the Er^3+^-doped NIR-IIb fluorescence probe to observe the tumors accumulation of nanodrug with 980 nm irradiation. To achieve real-time tumor retention, whole-body NIR-IIb imaging of nude mice was observed at different time points after immediately intravenously injected with LMDFP nanodrugs. Fluorescence intensity gradually increased at the tumor site as time extended from 0 to 8 h, and then gradually decreased (Fig. 3E). Meanwhile, NIR-IIb fluorescence imaging of all resected major organs and tumors further suggested that tumor tissue achieved peak accumulations at 8 h, which matched well with the results of in vivo imaging (Additional file 1: Fig. S19A, S19B). Additionally, we observed the Nd^3+^ ion content in major organs and tumor tissues to further evaluate the biodistribution of LMDFP using ICP-OES, which were consistent with that of the NIR-IIb imaging (Fig. 3F). LMDFP nanoparticles reached peak accumulations in tumor tissue at 8 h, according to the results. Therefore, we consider that 8 h post-injection of LMDFP is an optimal triggering time for PTT and DPA release.

**Fig. 4:**
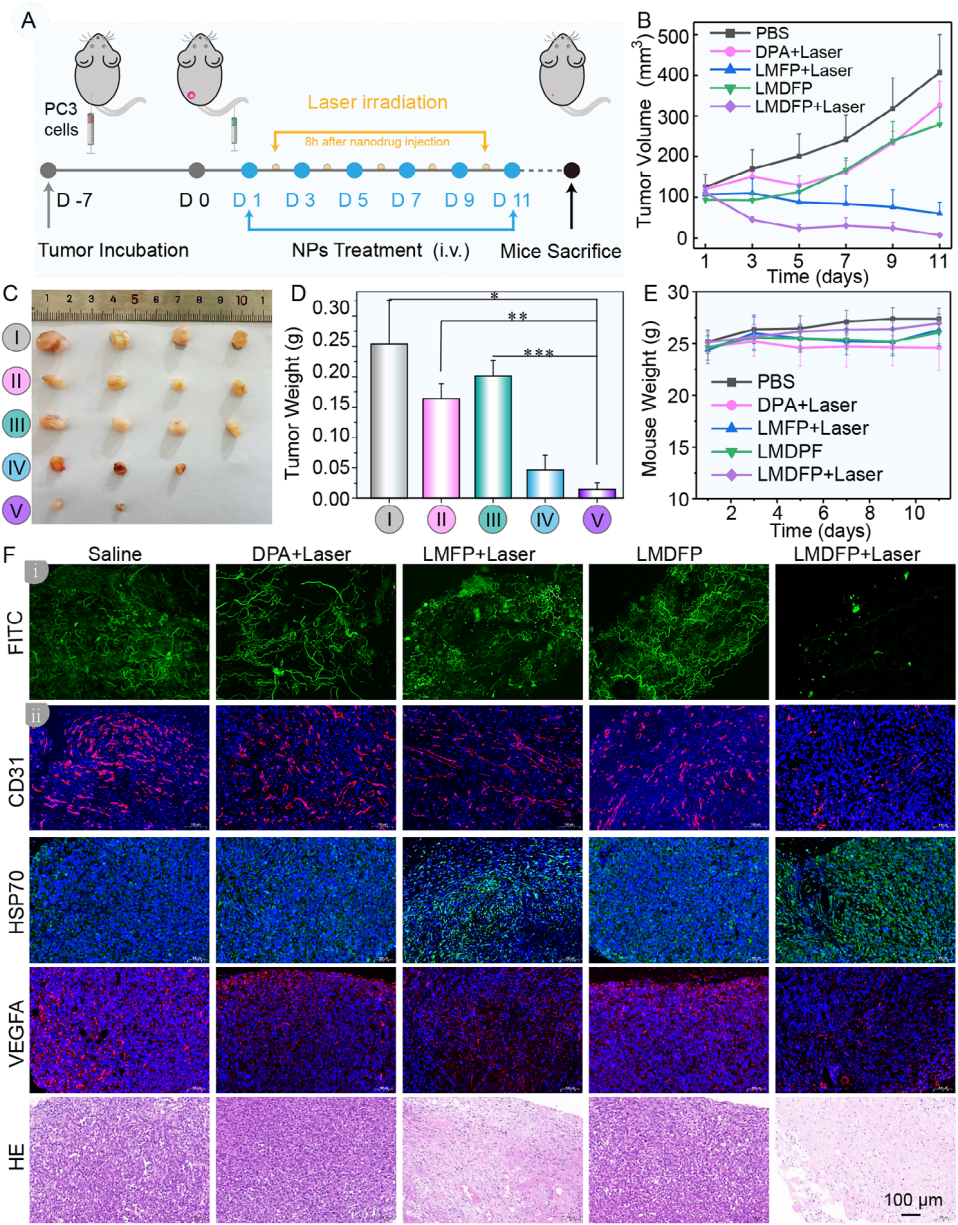
In vivo antitumor effect on PC-3 tumor loaded nude mouse model. (**A**) Schematic illustration of the therapeutic process in vivo (**B**) Tumor volume growth curves of five treatment groups in nude mice during the monitoring period. (**C**) Photographs of PC-3 tumors obtained from the nude mice after different treatments for 11 days. (**D**) Corresponding PC-3 tumor weight in the different treatment groups after 11 days (*P < 0.05, **P < 0.01, ***P < 0.001). (**E**) Body weight variation of nude mice after various treatments during the monitoring period. (**F**i) Tumor tissue vessel fluorescence imaging of FITC using CLSM. (**F**ii) CD31, HSP70, VEGFA expression and H&E staining analysis of PC-3 tumor tissues after the various treatments

Subsequently, the in vivo antitumor efficacy of LMDFP was further evaluated in PC-3 tumor-bearing mice. As is shown in Fig. 4A, the mice were randomly divided into five groups (*n* = 4) and intravenously injected with the following drugs every 2 days interval: (1) saline (100 µL), (2) DPA + Laser (100 µL, 5 mg/kg), (3) LMD (100 µL, 10 mg/kg), (4) LMFP + Laser (100 µL, 10 mg/kg), and (5) LMDFP + Laser (100 µL, 10 mg/kg). Then, mice in groups 2, 4, and 5 were exposed to 808 nm laser (1.5 W/cm^2^) at 8 h post i.v. injection for 5 min. The tumor volume and body weight of all groups were measured every 2 days and the tumors were collected on day 11. During the treatment period, the tumor volumes of nude mice were significantly suppressed in the LMFP + Laser and LMDFP + Laser groups, while the saline group showed a 4-fold increase in the tumor volume (Fig. 4B). The excised tumors were weighed and photographed after 11 days of treatment (Fig. 4C and D). The results indicated that the group treated with LMDFP + Laser exhibited the lightest tumor weight and smallest tumor size compared to other groups, which demonstrated the synergistic action of PTT and anti-angiogenesis. Furthermore, after the administration of various treatments, we labeled the blood flow with FITC to insight anti-vasculature effect in tumor tissue using CLSM for tumor blood vessel imaging. Compared to the control group, the vascular density of tumors at LMDFP + Laser treatment significantly decreased (Fig. 4Fi), which reflected that tumor vascular growth in LMDFP + Laser group was significantly inhibited. To further study the antitumor mechanism, immunofluorescence (IF) staining was carried out to demonstrate the histopathological changes of tumor tissue. As shown in Fig. 4Fii, CD31 labeled the blood vessels and VEGFA expression showed significant attenuation in LMDFP + Laser group, suggesting LMDFP nanodrugs strikingly anti-angiogenesis and tumor-targeting ability in these mice. As shown in Additional file 1: Fig. S20, the quantitative results of VEGFA expression were consistent with their fluorescence images. Notably, CD31 and VEGFA expression in LMFP + Laser group is mildly decreased, which may be due to the destruction of tumor tissue by PTT action and further affected the vascular formation. In addition, to evaluate the effect of PTT, HSP70 staining analysis was performed. As is shown in Fig. 4Fii, the HSP70 expression in LMFP + Laser and LMDFP + Laser groups was obviously increased, which indicates that photoinduced PTT was highly efficient in the tumor-bearing mice. Hematoxylin and eosin (H&E) staining shows significant vacuolation, nuclear shrinkage, and cell membrane rupture in the tumor tissue treated with LMDFP + Laser. These results collectively demonstrate that LMDFP are able to induce simultaneous anti-angiogenesis and PTT in tumor and show excellent antitumor ability in situ.

**Fig. 5:**
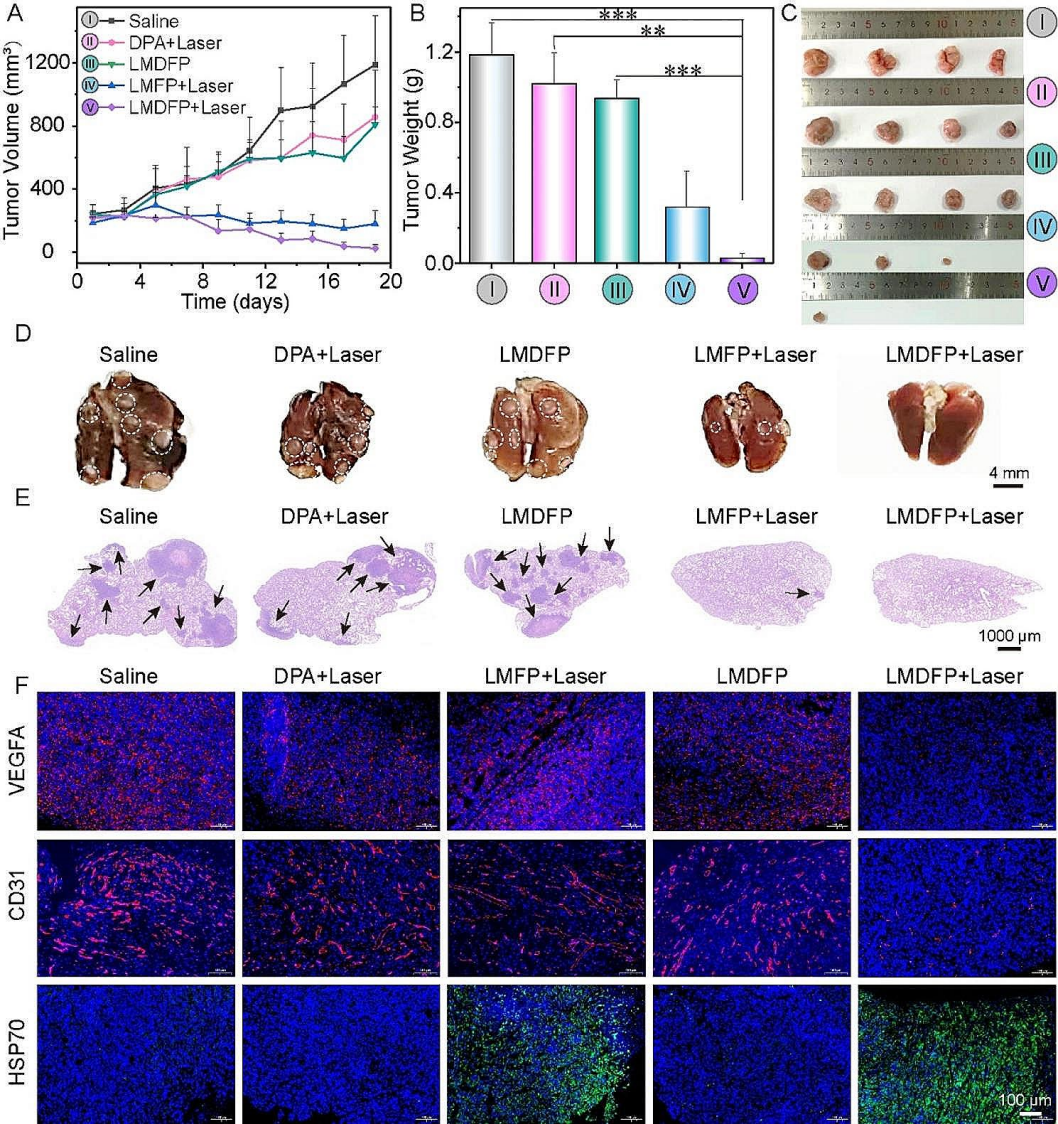
The antitumor metastasis study in vivo on 4T1 tumor loaded Balb/c mouse model. (**A**) Tumor volume growth curves of five treatment groups during the monitoring period. (**B**) Corresponding 4T1 tumor weight in the different treatment groups after 19 days (**P < 0.01, ***P < 0.001). (**C**) Photograph of 4T1 tumors obtained from the Balb/C mice after different treatments for 19 days. (**D**) Representative lung photographs collected from mice after various treatments. White nodules were metastatic tumors in the lungs. (**E**) H&E staining of lung metastatic lesions after different treatments. (**F**) VEGFA, CD31 and HSP70 expression of 4T1 tumor tissues after the various treatments

Tumor metastasis is a primary cause of cancer death [44]. Inhibiting tumor proliferation and metastasis can significantly extend the survival period of patient. LMDFP effectively inhibited PD-L1 expression and induced ICD, and then activated the antitumor immune response, contributing to tumor metastasis inhibition. Based on this, we evaluated the ability of LMDFP to inhibit lung metastasis of 4T1 tumor-bearing Balb/c model. Firstly, 4T1 tumor-bearing mice were randomly divided into five groups, and were treated with different materials every 2 days. After the injection of 8 h, the tumors were irradiated by 808 nm laser (1.5 W/cm^2^, 5 min) in DPA, LMFP and LMDFP groups. The tumor volume and body weight of all groups were measured every 2 days. During the treatment period, the *in-situ* tumor volumes were significantly suppressed in the LMFP + Laser and LMDFP + Laser groups, while the saline group showed a 6-fold increase in the tumor volume (Fig. 5A). After 18 days of treatment, the mice were euthanized and the tumor and lungs were collected. As is shown in Fig. 5B and C, the excised tumors were weighed and photographed, and the result is consistent with tumor volume growth curves. More importantly, a large number of metastatic nodes in the excised lungs of mice treated with saline were observed. On the contrary, the pulmonary metastasis nodules on lungs in LMFP + Laser and LMDFP + Laser groups were greatly suppressed. Notably, the sign of lung metastasis can hardly be observed in LMDFP + Laser (Fig. 5D, Additional file 1: Fig. S21). These results indicated that LMDFP + Laser can effectively inhibit the progression of lung metastasis, demonstrating that the synergized PTT and PD-L1 suppression action significantly enhanced antitumor immune responses. Because lung metastasis is a typical characteristic in breast cancer, we evaluated the lung metastasis level in various treated groups by representative H&E staining (Fig. 5E). Meanwhile, IF staining (CD31, VEGFA, and HSP70) of *in-situ* tumor was also carried out to further demonstrate the antitumor mechanism of LMDFP nanodrugs. Significant attenuated CD31 and VEGFA expression and increased HSP70 expression were observed in LMDFP + Laser group, suggesting the effective antiangiogenesis activity and PTT activity. This result was consistent with the observation in Fig. 5F. Above results of in situ tumor demonstrated that the LMDFP + Laser group exhibited more obvious tumor inhibition compared to the other groups, owing to the synergistic action of PTT and anti-angiogenesis to the tumor. All the above results indicated that LMDFP + Laser could effectively inhibit the proliferation and metastasis of 4T1 tumor in vivo.

**Fig. 6:**
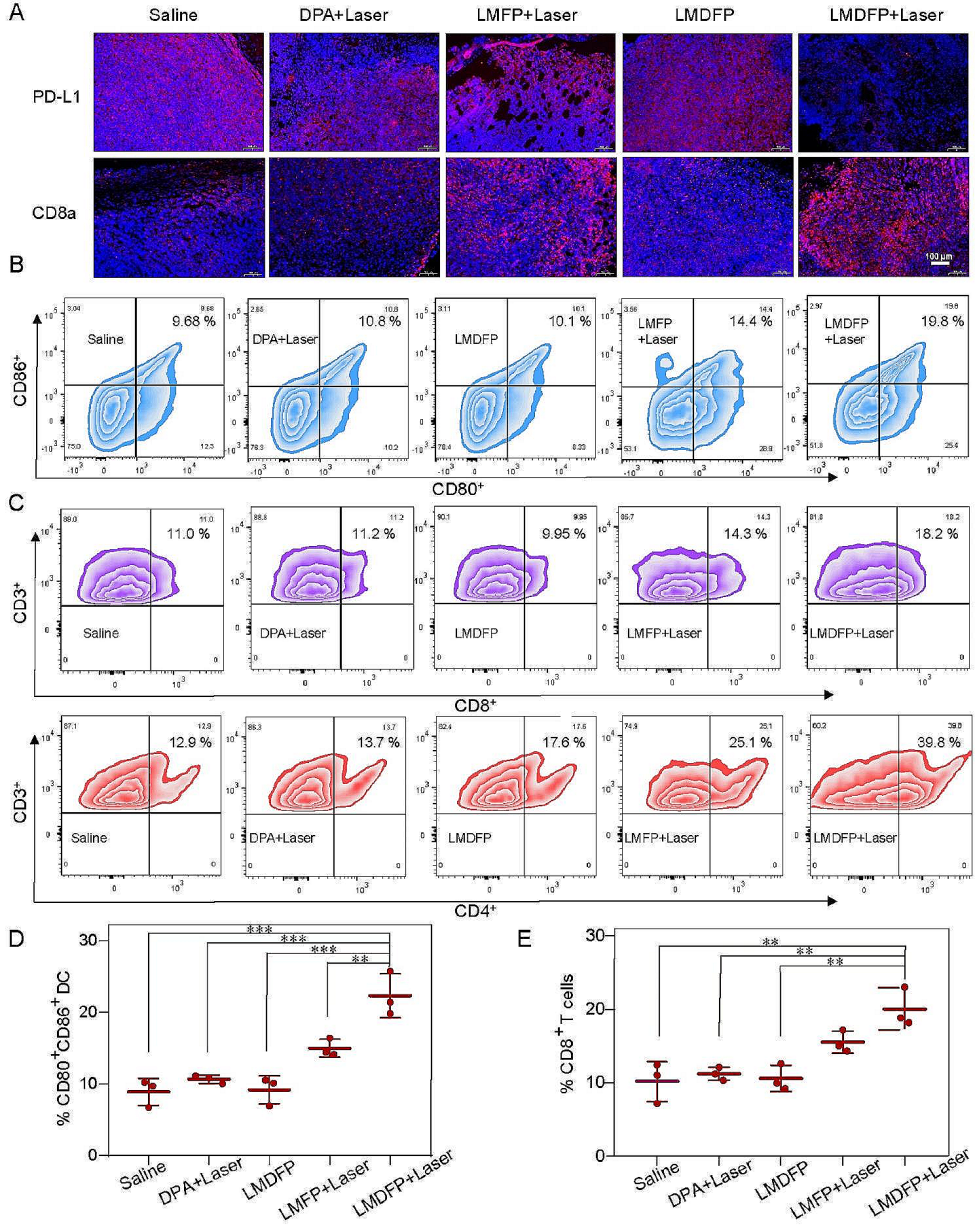
The antitumor metastasis study in vivo on 4T1 tumor loaded Balb/c mouse model. (**A**) PD-L1 and CD8a expression analysis of 4T1 *situ* tumor tissues after the various treatments. (**B, D**) Flow cytometry data of matured DC cells extracted from tumor tissues of mice-bearing 4T1 tumors (gated on CD11c^+^ DC cells). (**C, E**) Flow cytometry data of T lymphocytes extracted from tumor tissues after different treatments (first row: CD3^+^/CD8^+^; last row: CD3^+^/CD4^+^)

Moreover, we observed CD8a expression that plays an essential role in mature CD8 + T cells [45]. As depicted in Fig. 6A, LMFP + Laser and LMDFP + Laser groups display obvious upregulation of CD8a in IF staining of in-situ tumors. The results reflected that the PT effect in LMFP + Laser and LMDFP + Laser groups enhanced the expression of CD8a in tumor tissue. Furthermore, the LMDFP + Laser group shows the highest CD8a expression than other groups, it is because the inhibition of PD-L1 arising from DPA can further enhance the activation of T lymphocyte cells [46]. We next estimated the PD-L1 expression using IF staining. Due to the targeting and enhanced permeability and retention (EPR) effect of the tumor, the LMDFP delivered an amount of DPA molecules to the tumor site, which sharply inhibited PD-L1 expression through chelating copper ions in tumor (Fig. 6A). As DCs play a key role in the activation of the immune system, different treatments induced immunogenicity by assessing DC maturation and activation of T lymphocytes were evaluated. In order to evaluate immune response in vivo, 4T1 tumor-bearing mice were firstly treated with different drugs, then the tumors were excised and analyzed using flow cytometry assay after staining with CD11c, CD80 and CD86. The DC maturation was notably observed in LMDFP + Laser and LMFP + Laser groups as shown in Fig. 6B and D, which demonstrated that after the tumor is destroyed by PTT, DCs may be recruited to the damaged tumor site as antigen-presenting cells to trigger immune responses. To further evaluate whether LMDFP + Laser exerts an enhanced immunoregulatory effect, flow cytometry was employed to examine the activation of T lymphocytes, including assisted/induced (CD3^+^/CD4^+^) T lymphocytes and inhibitory/cytotoxic (CD3^+^/CD8^+^) T lymphocytes, which could combine with MHC to complete antigen presentation and immune response. As shown in Fig. 6C and E, more T lymphocytes were found in LMFP + Laser and LMDFP + Laser groups compared to control groups, illustrating effective immune activation after treatment in these mice. Among the experimental groups, LMDFP + Laser group displays much higher immunopotentiation than other groups, where the number of CD4^+^ T and CD8 + T cells is 49.5% and 22.4%, respectively. Subsequently, we evaluated the activation of CD4^+^ T and CD8^+^ T cells in the spleen of mice, which are consistent with the tumors (Additional file 1: Fig. S22). Together, these results demonstrate that LMDFP induced PTT can effectively activate the immune system and inhibit PD-L1 expression, blocking tumor immune escape and further enhancing the activation of T lymphocyte cells.

In order to further explore the biosafety of this therapeutic strategy, we examined the histology change of the major organs from 4T1 and PC-3 tumor-bearing mice after different treatment groups by H&E staining (Additional file 1: Fig. S23, S27). No significant histopathological abnormalities and signs of damage were found and no obvious difference in body weight was observed in 4T1 and PC-3 tumor-bearing mice models during therapy (Fig. 4E, Additional file 1: Fig. S24A), which suggests negligible side effects and systemic toxicity of LMDFP. Furthermore, pharmacokinetic evaluation was conducted after intravenously injected LMDFP (15 mg/kg). The results showed that LMDFP has a sufficient circulation time to perform antitumor action in the blood (Additional file 1: Fig. S19C), meanwhile, it could be timely excreted out from the body. Subsequently, to evaluate the biosafety, a blood routine examination was also conducted. According to the results depicted in Additional file 1: Fig. S25, various blood parameters such as red blood cell (RBC) count, mean corpuscular volume (MCV), mean corpuscular hemoglobin (MCH), platelet (PLT), hemoglobin (HGB), and mean corpuscular hemoglobin concentration (MCHC) did not show any significant changes among different groups after being intravenously injected with saline or LMDFP (24 and 48 h). In addition, the hemolysis assay showed that the LMDFP has excellent blood biocompatibility (Additional file 1: Fig. S26). The above results confirmed the excellent biosafety of LMDFP, showing the potential clinical application in cancer therapy. Taken together, our results demonstrate that the LMDFP nanodrug exhibits prominent PTT, anti-angiogenesis and inhibits tumor metastasis without obvious systemic toxicity.

## Conclusion

In summary, LMDFP copper nano-reaper with photothermal, antiangiogenic and initiated immune response effects has been constructed to enhance antitumor and restrain tumor metastasis. The nano-reaper is made up of downshifting emission and photothermal conversation LDNPs, DPA chelator loaded in mesoporous silica shell, and FA-PEG molecules outside. With the LDNPs, the nano-reaper can be endowed with both NIR to NIR downshifting luminescence for NIR-IIb bio-imaging and NIR to thermal for NIR light-triggered PTT. Meanwhile, the release of DPA chelator triggered by the photothermal effect exhibits excellent copper selection, which can effectively eliminate copper ions in tumor cells, downregulate PD-L1 expression, and inhibit tumor angiogenesis. Moreover, ICD induced by PTT and PD-L1 inhibition triggered by DPA copper chelator collectively enhance antitumor immune response to suppressive distant tumor metastasis. Overall, the LMDFP copper nano-reaper for tumor-specific PTT and anti-angiogenesis therapy demonstrates prominent tumor growth and metastasis suppression efficacy without obvious systemic toxicity and provides a new strategy for synergistic therapy.

### Electronic supplementary material

Below is the link to the electronic supplementary material.

Supplementary Material 1

## Data Availability

The datasets and materials used in the study are available from the corresponding author.
