# Supplementary material for: NIR-IIb fluorescence antiangiogenesis copper nano-reaper for enhanced synergistic cancer therapy
Source: J Nanobiotechnology. 2024 Feb 19;22:73. doi: 10.1186/s12951-024-02343-5 (PMC10877799; doi:10.1186/s12951-024-02343-5)
Supplement: Supplementary file 1 — Supplementary Material 1 [file 12951_2024_2343_MOESM1_ESM.docx]

**Supplementary Materials**

**NIR-IIb Fluorescence Antiangiogenesis Copper** **Nano-reaper for Enhanced Synergistic Cancer Therapy**

Wenling Li^1^, Huan Xin^1^, Wenjuan Gao, Pengjun Yuan, Feixue Ni, Jingyi Ma, Jingrui Sun, Jianmin Xiao, Geng Tian,* Lu Liu,* Guilong Zhang*

School of Pharmacy, Shandong Technology Innovation Center of Molecular Targeting and Intelligent Diagnosis and Treatment, Binzhou Medical University, Yantai 264003, P.R. China

^1^ Co-first author.

*** Corresponding Authors.

E-mail: [tiangengbmu@163.com;](mailto:tiangengbmu@163.com;) [luliu@bzmc.edu.cn](mailto:luliu@bzmc.edu.cn); [glzhang@bzmc.edu.cn](mailto:glzhang@bzmc.edu.cn)

**Experimental section**

**Materials.** All the reagents were used as received without further purification. Oleic acid (OA), 1-octadecene (ODE), Tetraethyl orthosilicate (TEOS), 1-pentanol, D-penicillamine (DPA) and fluorescein isothiocyanate (FITC) were acquired from Aladdin Reagents Co. Ltd. (Shanghai, China). Sodium hydroxide (NaOH), Ammonium fluoride (NH4F), Cetyltrimethylammonium bromide (CTAB) and Ammonia solution (NH_3_H_2_O) were obtained from the Sinopharm Chemical Reagent Co. Ltd. (Shanghai, China). YCl_3_·6H_2_O, YbCl_3_·6H_2_O, GdCl_3_·6H_2_O, ErCl_3_·6H_2_O and NdCl_3_·6H_2_O were purchased from Hwark Chem Co. Ltd. (Beijing China). FA-PEG3400 was purchased from Qiyue Biotech Co. Ltd. (Xi’an China). Vascular endothelial growth factor A (VEGFA) and interleukin-8 (IL-8) ELISA kits were purchased from ABclonal Biotechnology Co. Ltd (Wuhan China). Deionized water was produced by Heal Force pure water system (CR-SP412, China). All aqueous solutions are prepared in deionized water.

**Gd-OA (0.10 M) host** **precursor.** The precursor solution was synthesized according to previously reported. Briefly, the Gd-OA precursor was synthesized with the following protocol: 6 mmol of GdCl_3_·6H_2_O, 24 mL of oleic acid (OA) and 36 mL of octadecene (ODE) were magnetically mixed under vacuum to remove water and oxygen. The resulting mixture solution was heated to 130 °C under vacuum for 1 h. Finally, a clear and transparent Gd-OA precursor solution was obtained.

**Nd-OA (0.10 M) precursor.** The synthesis method of Nd-OA is similar to the synthesis procedure of Gd-OA precursor, only the corresponding rare earth chloride kinds need to be changed.

**Na-TFA-OA (0.40 M) precursor.** Briefly, 20 mmol of sodium trifluoroacetate (Na-TFA) and 50 mL of OA were magnetically mixed and heated to 60 °C for 1 h under vacuum. After that, Na-TFA-OA precursor solution was obtained.

**Synthesis of** **NaYF_4_:25%Yb:2%Er****.** According to previously reported method [1]. YCl_3_·6H_2_O (0.73 mmol), YbCl_3_·6H_2_O (0.25 mmol), ErCl_3_·6H_2_O (0.02 mmol), OA (6 mL) and ODE (15 mL) were magnetically mixed under vacuum and heated to 130 °C for 1 h. After cooling to 50 °C, 8 mL of methanol solution containing 2.5 mmol of NaOH and 4 mmol of NH_4_F was added to keep for 1 h. The resulting mixture solution was slowly heated to 100 °C under vacuum to remove methanol, before heated to 300 °C for 1 h under argon gas. Anhydrous ethanol was added to precipitate the nanocrystals, and the product was washed with ethanol for at least 3 times. Finally, the precipitated nanocrystals were redispersed in 10 mL of cyclohexane for further use.

**Synthesis of** **NaYF_4_:25%Yb:2%Er @NaGdF_4_ core@shell nanoparticles.** A layer of NaGdF_4_ was grown over the NaYF4:25%Yb:2%Er nanocrystal core using a continuous layer-by-layer growth method. 0.25 mmol of NaYF4:25%Yb:2%Er cyclohexane solution was dissolved in the mixture of OA (4 mL) and ODE (6 mL) in a three-neck flask. The mixture solution was heated to 100 °C to remove cyclohexane and then was heated to 280 °C before 7.5 mL of 2:1 mixed precursor solution of Gd-OA (0.10 M) and Na-TFA-OA (0.40 M) was added dropwise. The resulting mixture solution was aged for 1 h at 280 °C under argon atmosphere. After cooling to room temperature, the product was washed with ethanol for at least 3 times. Finally, the precipitated nanocrystals were redispersed in 2.5 mL of cyclohexane for further use.

**Synthesis of** **NaYF_4_:25%Yb:2%Er@NaGdF_4_@NaNdF_4_ nanoparticles.** The synthesis process of core-shell structure NaYF_4_:25%Yb:2%Er@NaGdF_4_@NaNdF_4_ (LDNP) is similar to the preparation of NaYF_4_:25%Yb:2%Er @NaGdF_4_ nanocrystals except that Nd-OA (0.10 M) and Na-TFA-OA (0.40 M) shell precursors were alternately introduced by dropwise addition.

**Synthesis of LDNP@mSiO_2_.**According to previously reported method [2]. 1 mL of LDNPs cyclohexane solution were uniformly dispersed into 10 mL of cyclohexane solution containing 0.44 mL of n-pentanol, and then 1 mL of TEOS was added into the mixed solution. After stirring for 30 min at room temperature, 20 mL of water containing 1.0 g of CTAB and 0.3 g of urea was added to keep for 16 h at 70 °C in an oil bath. Finally, the solution was washed with ethanol and water five times. The residual CTAB in the LDNP@mSiO_2_ was extracted by redispersed in 1% HCl ethanol solution and the mixture solution was refluxed at 60 °C for 7 h 3 times.

**Synthesis of LDNP@mSiO_2_-DPA@FA-PEG.** 0.3 g of D-penicillamine was sonicated in 30 mL of water, and then 2 mL of LDNP@mSiO_2_ solution removed CTAB was added. After stirring for 12 h at room temperature, 200 mg of FA-PEG3400 was added to the above solution for 24 h. Finally, the product LDNP@mSiO_2_-DPA@FA-PEG was washed with deionized water for 3 times.

**Photo-thermal investigation.** The PT property of LMDFP nanoparticles were examined using an infrared thermal imaging system (Fotric 226s-L28/1). 1 mL of LMDFP nanoparticles with different concentrations at 100, 200, 400, 800 μg/mL in 1.5 mL centrifuge tube were irradiated using 808 nm laser (LSR-PS-ll5) with the power density of 1.0 W/cm2 for 10 min. In addition, the PT properties of LMDFP at the concentrations of 200 μg/mL under different laser power density (0.5, 1, 2, 4 W/cm^2^) were measured through the similar procedure, respectively. To study the PT stability, 1 mL of LMDFP at 200 μg/mL was introduced into a centrifuge tube and irradiated with 808 nm laser at the power density of 2.0 W/cm^2^ for 5 min. After cooling down to the ambient temperature, the irradiation procedure started again and repeated for 5 times.

**Calculation of the PT conversion efficiency.** The PT conversion efficiency of LMDFP NPs was determined according to a previous method. Briefly, 1 mL of LMDFP NPs (200 μg/mL) were introduced into a centrifuge tube and irradiated under a 808 nm laser (2.0 W/cm^2^) for 5 min. The temperature was recorded until the cool down to ambient temperature. The PT conversion were calculated according to the method as follows:

η=$\frac{hA( T_{max}-T_{amb}) - Q_{0}}{I(1 - {10}^{-A\lambda})}$ (1)

where η is the PT conversion, h is the heat transfer coefficient, A is the surface area of the container, T_max_-T_amb_ is defined as the temperature change (T_max_ is the maximum temperature of the solution and T_amb_ is the ambient temperature of the surroundings, respectively.) Q_0_ is the heat associated with the light absorbance of the solution, 1 is the laser power density, Aλ is the absorbance of the LMDFP NPs at the wavelength of 650 nm. The value of hA can be calculated via the following equation:

ℎ𝐴 =$\frac{m_{D} \times C_{D}}{\tau_{s}}$ (2)

where m_D_ and C_D_ (4.2 J g^-1^) is the mass and heat capacity of the solvent (DI water), τ_s_ is the PT conversion efficiency from solution to the surroundings, which can be obtained from the cooling period.

**Evaluation of copper-chelating ability of DPA.** DPA (100 µL, 10 mg ml^-1^) was added to tubes containing 2 ml various metal ions solution. The mixtures were shaken at 37 °C for 12 h, and then filtered by Amicon Ultra centrifugal filters. The filtrates were analyzed by ICP-OES to determine the remaining metal content. The amount of metal ion adsorbed by DPA was calculated by subtracting the residual metal ion concentration from the initial concentration.

**In vitro cell cytotoxicity evaluation.** PC3 (human prostate cancer cells), 4T1 (murine breast cancer cells), THLE-3 (human liver epithelium cells) and 293T (human renal epithelial cells) were respectively seeded in 96-well culture plates at a density of 1×10^4^ per well at 37 °C under 5% CO_2_ overnight. Subsequently, the cells were treated with different nanodrugs at varied concentrations (5 μg/mL, 10 μg/mL, 20 μg/mL, 40 μg/mL, 80 μg/mL, 160 μg/mL and 200 μg/mL). After incubation for 8 h, the groups of DPA and LMDFP groups in 4T1 and PC3 lines were irradiated by 808 nm of laser with energy power density of 1.5 W/cm2 for 5 min, respectively. After co-incubation for total 24 h or 48 h, the cells were washed with fresh PBS and the cell viability was evaluated using cell-counting kit-8 (CCK-8) assay.

**Live-dead cell staining experiments.** PC3 cells were planted into 6-well culture plates at a density of 1 × 10^6^ cells per well and incubated at 37 °C incubator overnight. Afterwards, PC3 cells were incubated with saline, DPA (50 μg/mL), LMD (200 μg/mL), LMDFP (200 μg/mL) for 8 h. Then, the groups of DPA+Laser and LMDFP+Laser were irradiated by 808 nm laser at 1.5 W/cm2 for 5 min, respectively. Finally, after incubated with different treatments for 24 h, 4T1 cells were stained with calcein-AM (green, live cells) and pyridine iodide (PI) (red, dead cells) staining reagents to observe the viable and dead cells using a confocal fluorescence microscope (LSM-800, Zeiss, Germany).

**Transwell assay.** To observe migration of 4T1 and PC3, the cells were seeded into the upper chambers at a density of 5×10^4^ cells well^-1^ and cultured in media containing DPA (50 µg/mL), LMD (200 µg/mL) and LMDFP (200 µg/mL), respectively. Then, the cells were allowed to migrate for 24 h toward the lower chambers filled with culture media containing 10% FBS, where the groups of DPA+Laser and LMDFP+Laser were irradiated by 808 nm laser at 1.5 W/cm^2^ for 5 min at 4 h of incubation. Next, the cells that migrated to the bottom side of the membranes were fixed with methanol for 5 min and stained with crystal violet for 15 min. In invasion experiment, 50 uL of matrigel matrix was added to the upper chambers at 37 ℃ for 1 h to allow solidification. The subsequent steps were similar to above cell migration process.

**Tube-formation assay.** C166 (Mouse vascular endothelial cells) cells at a density of 1×10^5^ cells well^-1^ were seeded into six-well plates and cultured at 37 ℃ for 12 h. After cell adhesion, 2 mL of DPA (50 μg/mL), LMD (200 μg/mL) and LMDFP (200 μg/mL) media solution were added. After incubation of 8 h, DPA + Laser and LMDFP + Laser groups were irradiated by 808 nm laser at 1.5 W/cm^2^ for 5 min, respectively. Finally, after incubated with different treatments for 48 h, cells were collected and seeded in 96-well added 50 μL of matrigel fixed at 37 ℃ for 1h in advance. The endothelial cell tubes were imaged and counted every 1 h.

**In vitro cytokine measurement.** PC3 cells were plated at a density of 1×10^5^ cells in 6 well plate and incubated for 24 h. Cells were then treated with saline, DPA (50 µg/mL), LMD (200 μg/mL) or LMDFP（200 μg/mL）for 8 h. Then, the groups of DPA+Laser and LMDFP+Laser were irradiated by 808 nm laser at 1.5 W/cm^2^ for 5 min, respectively. Finally, after incubated with different treatments for 24 h. Conditioned media was collected, centrifuged for 5 min at 2000 rpm, and divided into 100 µL aliquots. The amounts of VEGFA and IL-8 released in the media were measured by VEGF ELISA kit and IL-8 ELISA kit.

**Western blot analysis.** PC3 cells were incubated with saline, LMDFP at a dosage of 200 μg/mL for 4 h. Then, the groups of LMDFP + Laser was irradiated by 808 nm laser at 1.5 W/cm^2^ for 5 min. Finally, after incubated with different treatments for 12 h. The cells washed with cold fresh PBS, corresponding protein was extracted from PC3 cells using the total protein extraction kit and the protein concentrations were determined by BCA protein assay kit. Afterwards, the proteins were separated by SDS-polyacrylamide gel electrophoresis and transferred to polyvinylidene difluoride membranes. Next, selected antibodies were incubated with membranes overnight at 4 °C. Finally, protein expression could be observed using the chemiluminescence system.

**qRT-PCR detection.** PC3 cells were seeded into the 12-well plates and then incubated with saline, LMDFP at the concentration of 200 μg/mL for 4 h. Then, the groups of LMDFP + Laser was irradiated by 808 nm laser at 1.5 W/cm^2^ for 5 min. Finally, after incubated with different treatments for 12 h. The total RNA was extracted using trizol reagent. Next, cDNA was synthesized and analyzed. The primers were used as following:

HSP70-Foward,

5′GCGGAGATAACGGAGGAGATG;

HSP70-Reverse,

5′AGTCTCGGATGGCAGCATTTG;

VEGFA-Foward,

5′AGGGCAGAATCATCACGAAGT;

VEGFA-Reverse,

5′AGGGTCTCGATTGGATGGCA;

E-cadherion-Foward

5′CGAGAGCTACACGTTCACGG;

E-cadherion- Reverse

5′GGGTGTCGAGGGAAAAATAGG;

**In vivo Anti-tumor Effect.** All animal experiments were studied in accordance with the rules approved by the animal ethics committee of Binzhou Medical University. 4T1 cells of 1×10^6^ and PC3 cells of 1×10^7^ were injected subcutaneously on the right sides of Balb/c and nude mice, respectively. When the tumor volume of PC3 tumor reached approximately 100 mm^3^ or 4T1 tumor volume reached approximately 200 mm^3^, the mice were divided into five groups: (a) Saline; (b) DPA + laser; (c) LMDFP; (d) DMFP + Laser; (e) LMDFP + Laser. After that, the tumor loaded mice were injected intravenously with Saline, DPA (2 mg/kg), DMFP or LMDFP at 8 mg/kg every alternate day. For group (b), (d) and (e), the cancer bearing mice were irradiated with 808 nm laser (1.5 W/cm^2^) for 5 min at 8 h post-injection. Isoflurane was used for anesthesia of the mouse during irradiation. The tumor volume and body weight were recorded every 2 days, and tumor volumes could be recorded using a formula: V=a×b^2^/2. where “a” and “b” were the longest and shortest diameters of the tumor, respectively. After 11- or 18-days’ treatment, tumors as well as main organs were collected from sacrificed mice for further immunohistochemistry assay, histopathology evaluation and immune cells extraction.

**Flow Cytometry Analysis.** Tissue fractions homogenates obtained from above tumor were digested with 1 mg mL^-1^ of collagenase for 1 h at 37 °C and then filtered through a cell strainer to obtain the cells. These cells were resuspended in PBS buffer to get single-cell suspension. To study the in vivo DC maturation, the cells were stained with CD11c-Pacific Blue-A, CD80-FITC, and CD86-APC antibodies at room temperature for 30 min in the dark according to the manufacturer’s protocols. Stained cells were detected by flow cytometer and analyzed using FlowJo software. For the analysis of infiltrated T lymphocytes in tumors, the cells were stained with CD3-APC, CD4-PE, and CD8-PerCP-Cy5.5 antibodies for flow cytometry.

# References

# Li X, Shen D, Yang J, Yao C, Che R, Zhang F, Zhao D. Successive layer-by- layer strategy for multi-shell epitaxial growth: shell thickness and doping position dependence in upconverting optical properties. Chem Mater. 2012;25(1):106-112.

# Dai Y, Yang D, Yu D, Xie S, Wang B, Bu J, Shen B, Feng W, Li F. Engineering of monodisperse core-shell up-conversion dendritic mesoporous silica nanocomposites with a tunable pore size. Nanoscale. 2020;12(8):5075-5083.


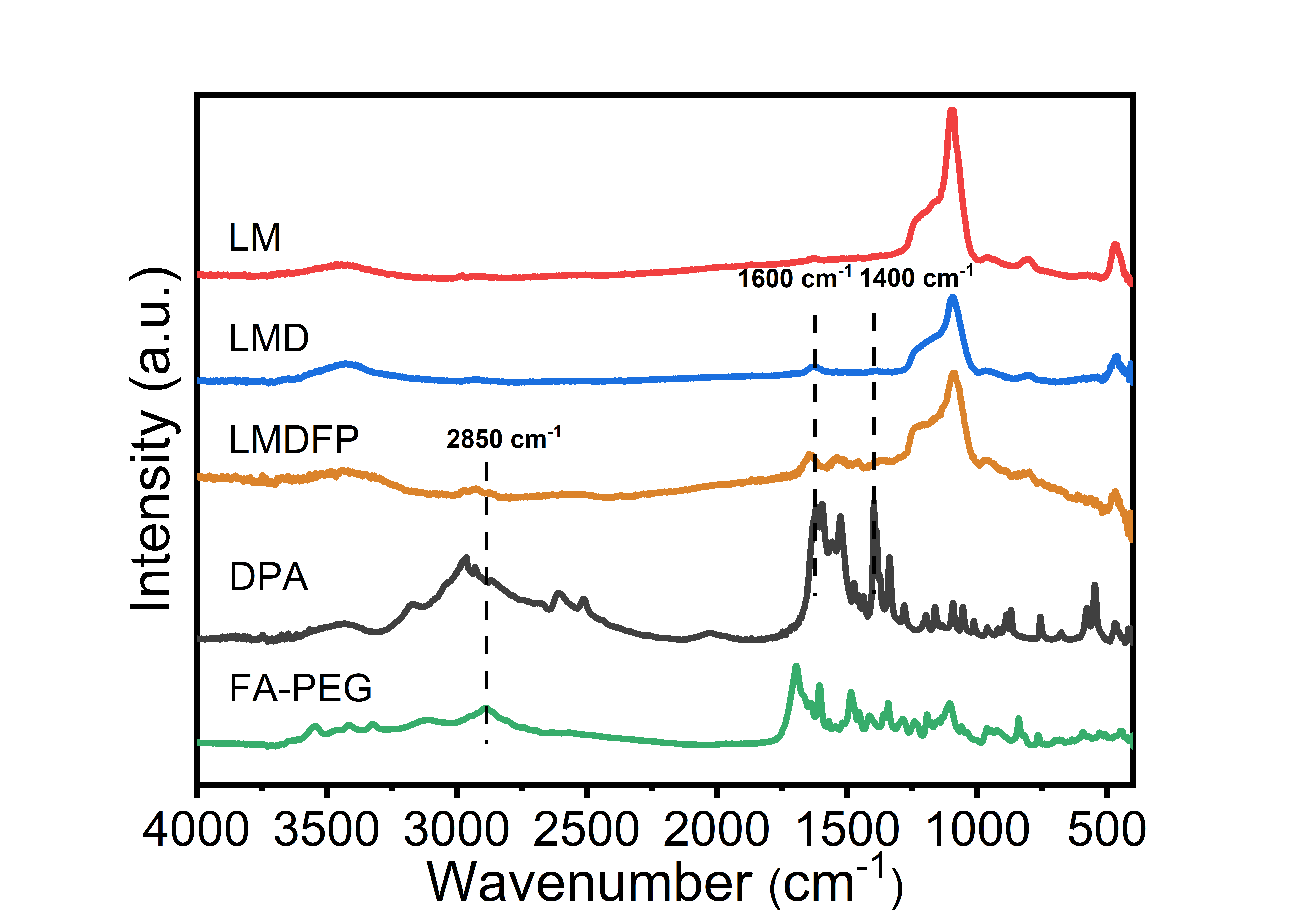


**Fig. S1.** Fourier transform infrared (FT-IR) spectra of FA-PEG-coated LMDFP nanoparticles and LMD nanoparticles.


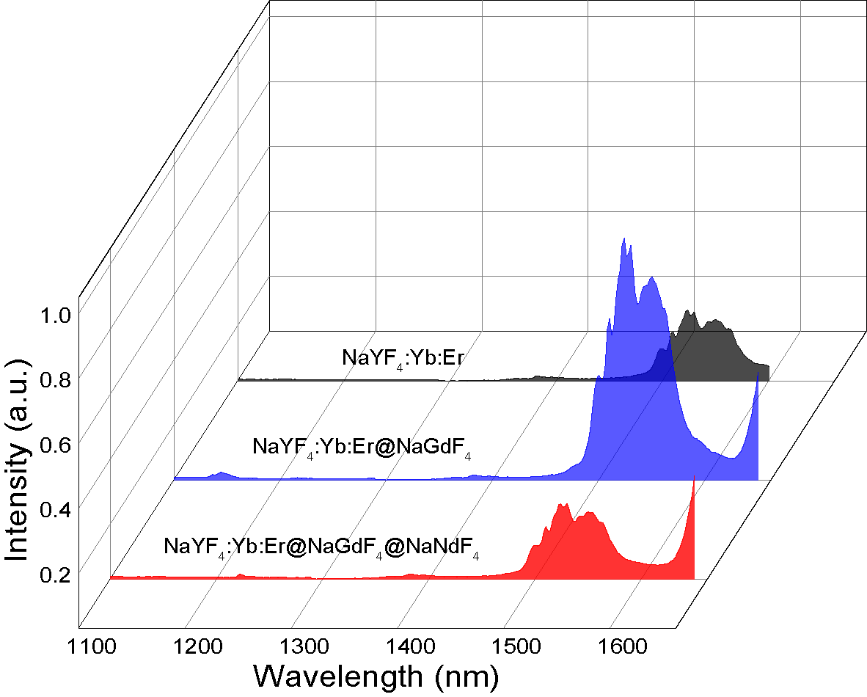


**Fig. S2.** Upconversion emission spectra of NaYF_4_:Yb,Er with different shell.


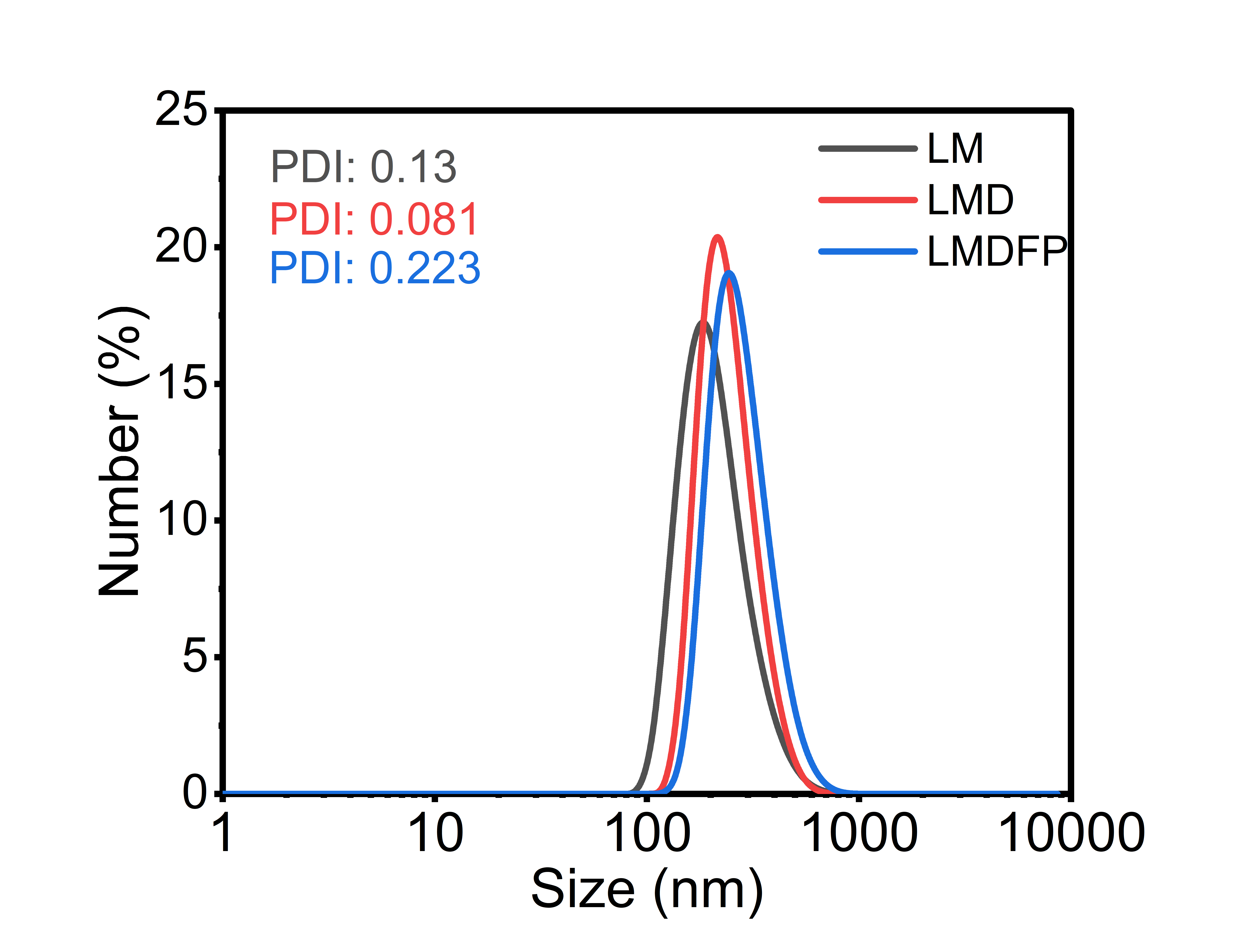


**Fig. S3.** Hydrodynamic radii and polydispersity (PDI) values of the particles calculated from the DLS autocorrelation functions.


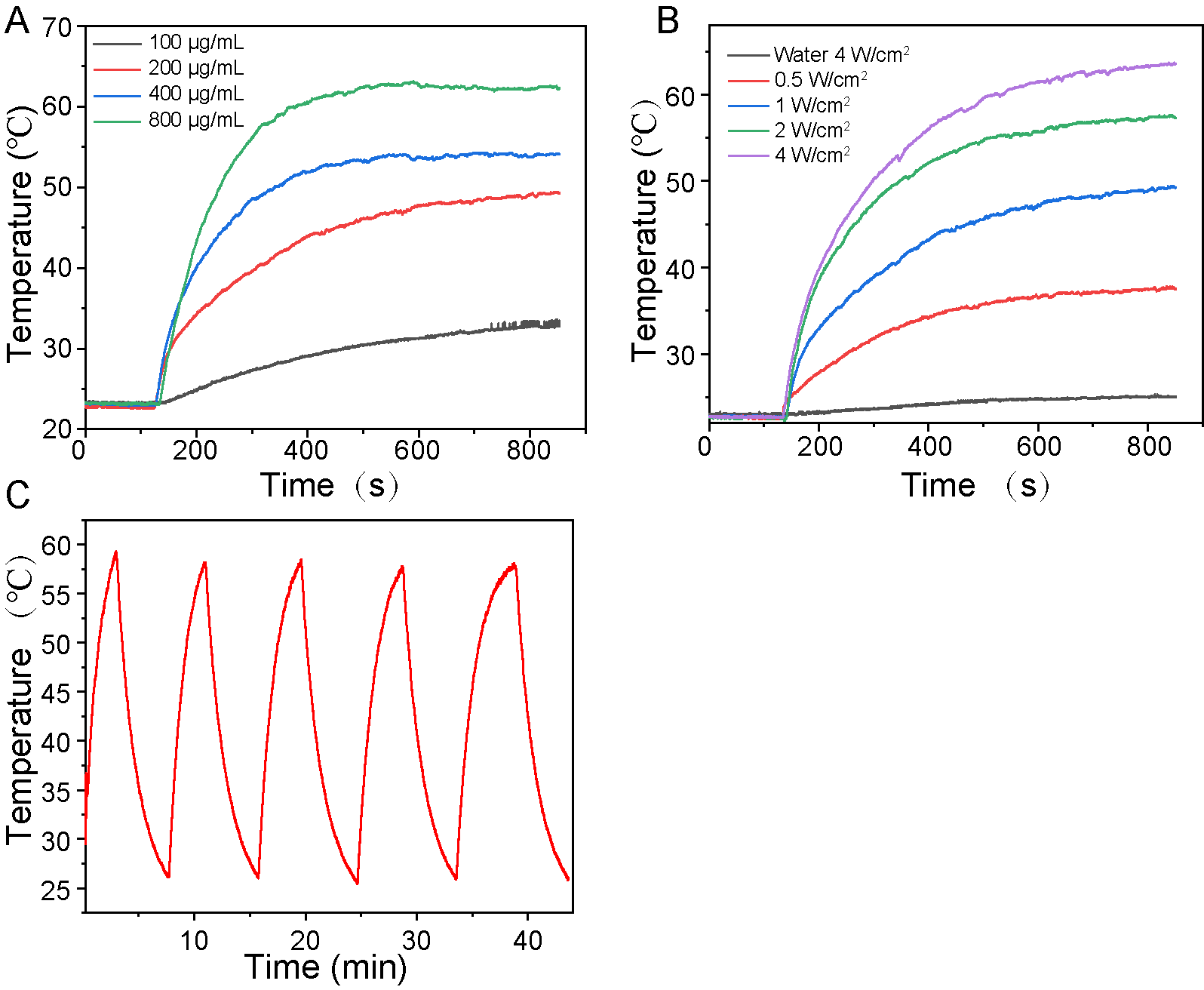


**Fig.** **S4.** (A)The temperature change of LMDFP with different solution concentration under 808 nm laser irradiation, respectively. (B) the temperature change of LMDFP solution irradiated by different power densities of 808 nm laser for 10 min, respectively. (C) the photothermal stability of LMDFP nanoparticles is observed by irradiation for five cycles under 808 nm laser with a power density of 2 W/cm^2^.


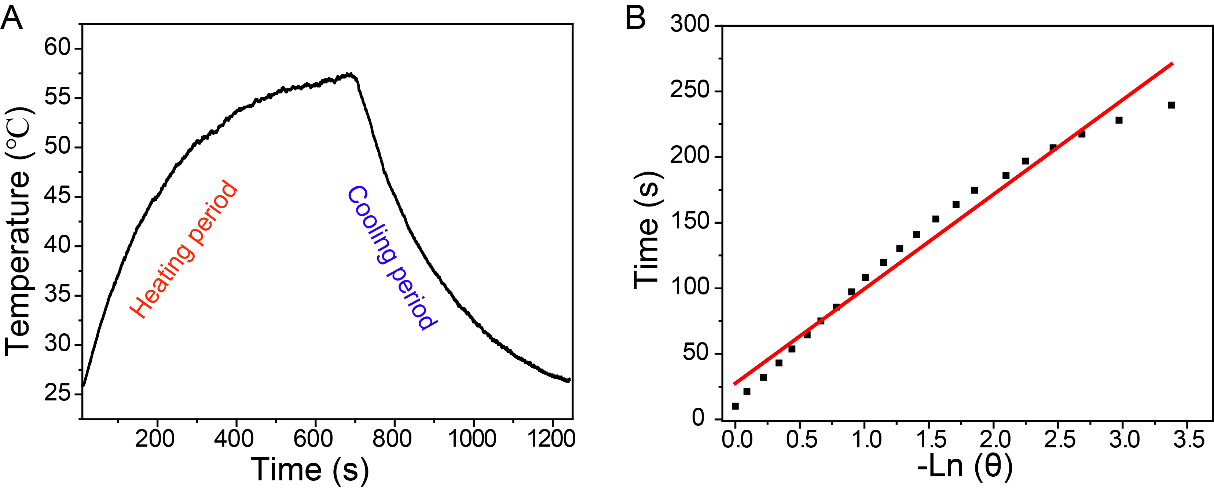


**Fig. S5.** (A) The heating and cooling curves of the LMDFP. (B) Fitted curve of photothermal conversion efficiency of LMDFP.


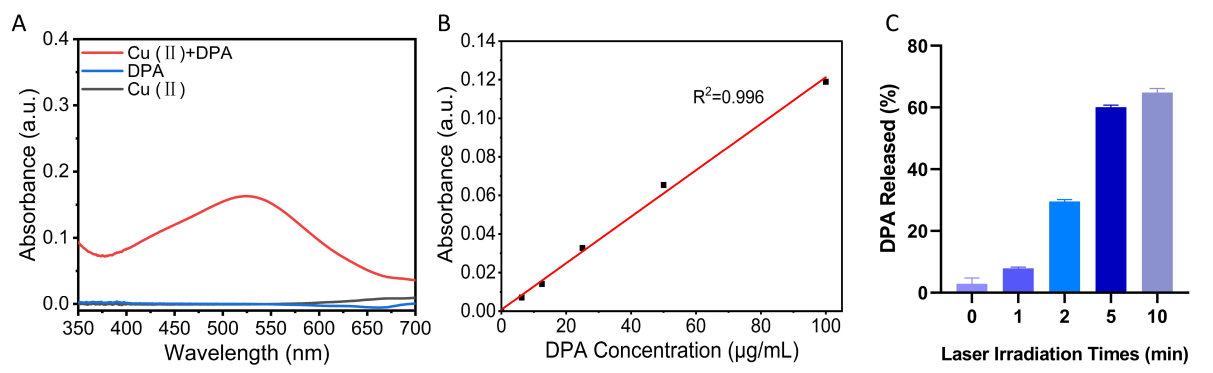


**Fig. S6.** DPA controllable photothermal release from the nano-reaper LMDFP. (A) UV absorption spectra of copper ion chelated DPA. (B) The standard curve of copper ion chelating different concentrations of DPA. (C) The release behavior of DPA in LMDFP under different laser irradiation times.


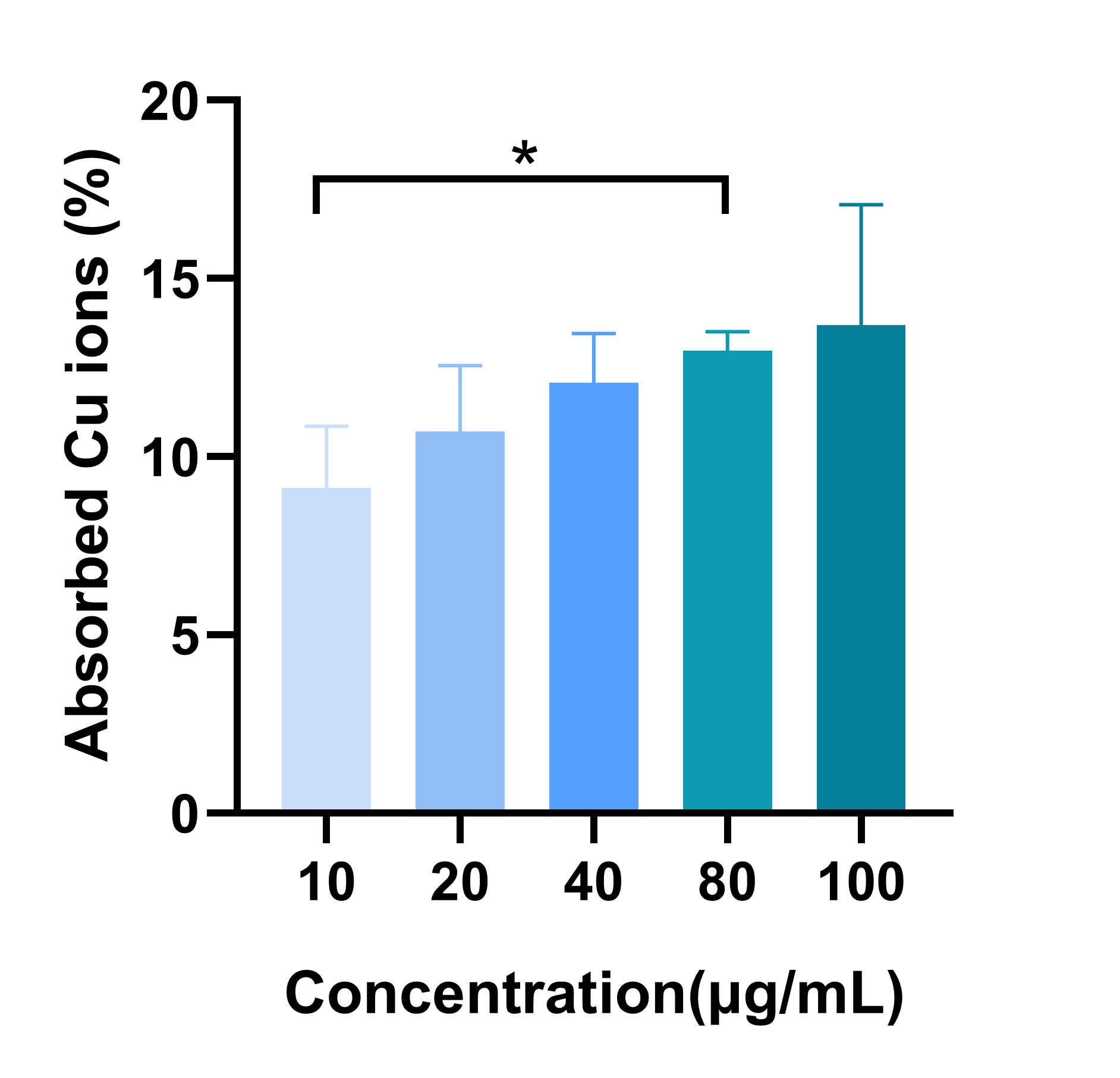


**Fig. S7.** The chelating capacity of LMDFP to copper ions. At higher concentrations of copper ions nano-reaper LMDFP, the intensity of absorbed Cu ions significantly increases due to the copper depletion ability of DPA released from LMDFP nanodrug.


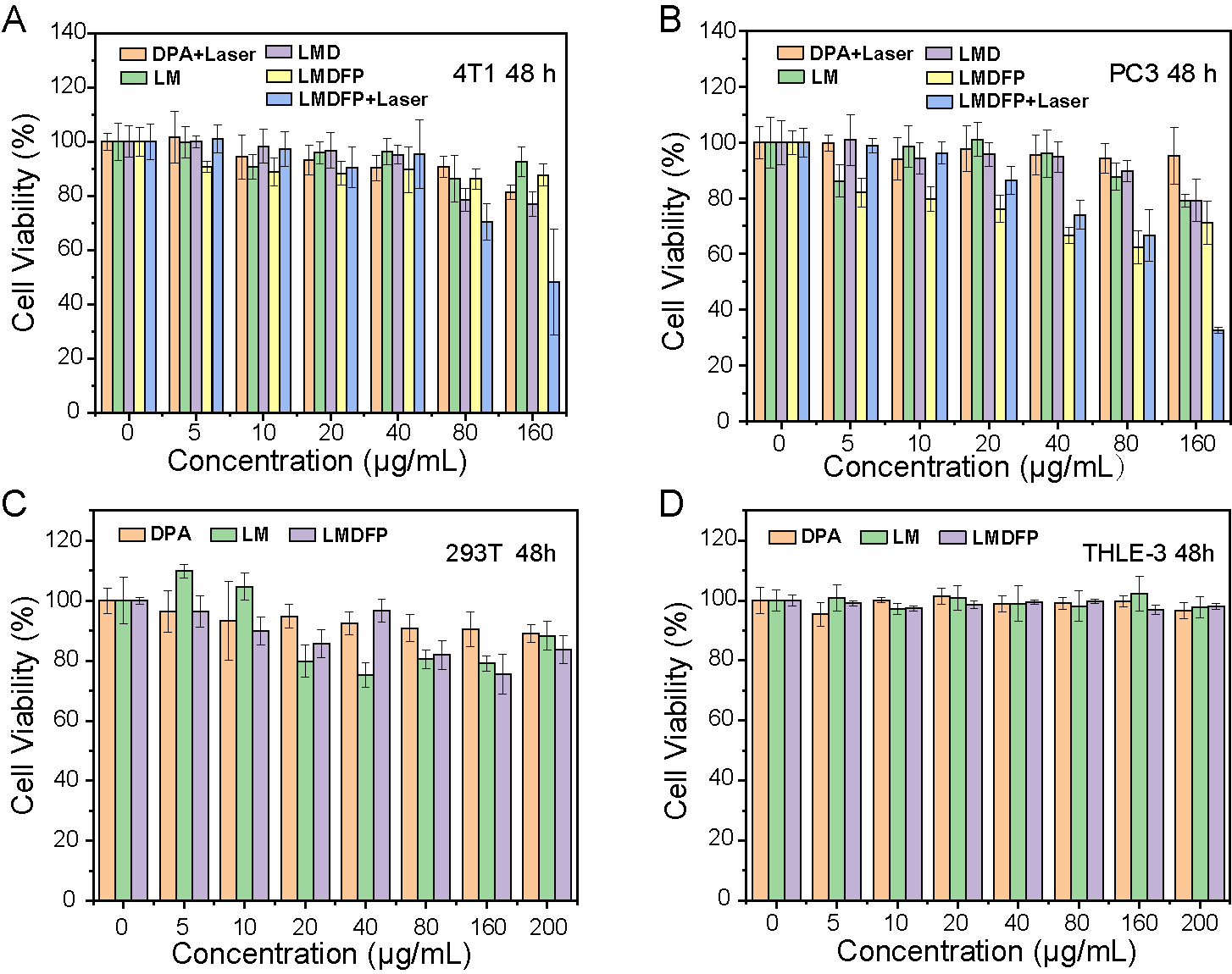


**Fig. S8.** Relative cell viability of (A) 4T1 (B) PC3 (C) 293T and (D) THLE-3 cells after incubation with different nanoparticles at various concentrations for 48 h.


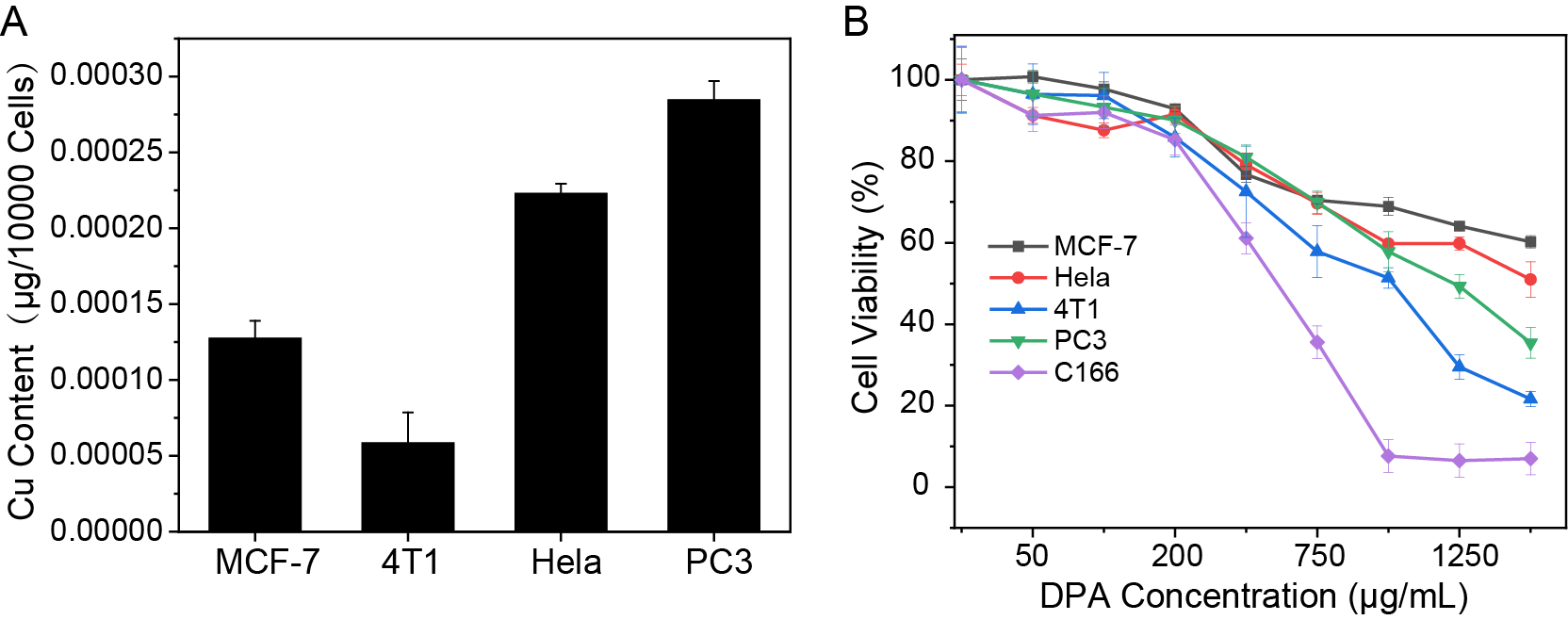


**Fig. S9.** (A) Cu content in different tumor cells. (B) Relative cell viability of MCF-7, HeLa, 4T1, PC3 and C166 cells after incubation with DPA at various concentrations for 24 h.


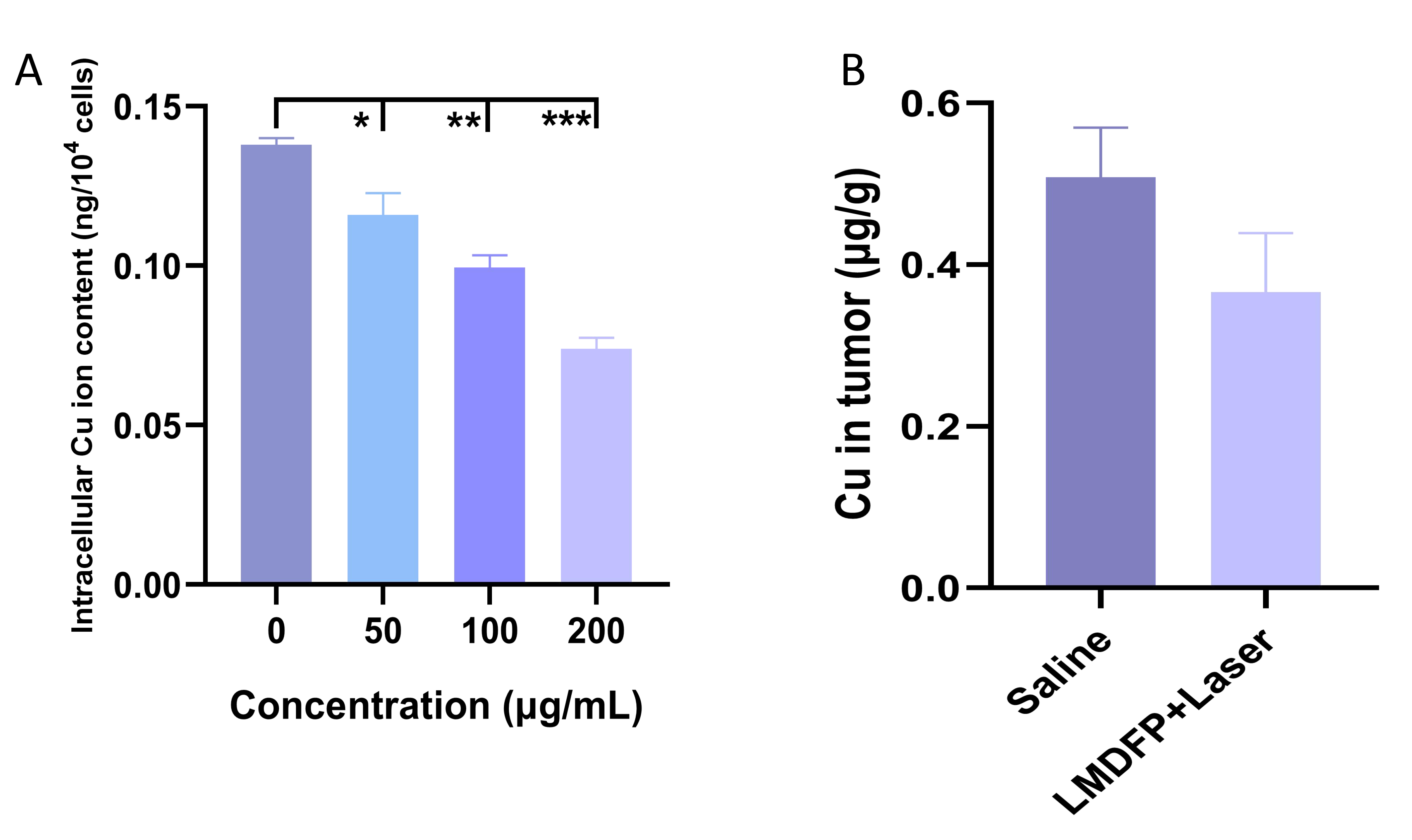


**Fig. S10.** (A) The chelating capacity of LMDFP to copper ions in 4T1 cancer cells. (B) The chelating capacity of LMDFP to copper ions in mice bearing 4T1 tumors. For 4T1 cancer cells, at higher concentrations of copper ions nano-reaper LMDFP, the concentration of Cu ions decreases due to the copper depletion ability of DPA released from LMDFP nanodrug. In the copper ions nano-reaper LMDFP + Laser treated 4T1 bearing mice, the concentration of Cu ions decreased due to the copper depletion ability of DPA released from LMDFP nanodrug under 808 nm laser irradiation.


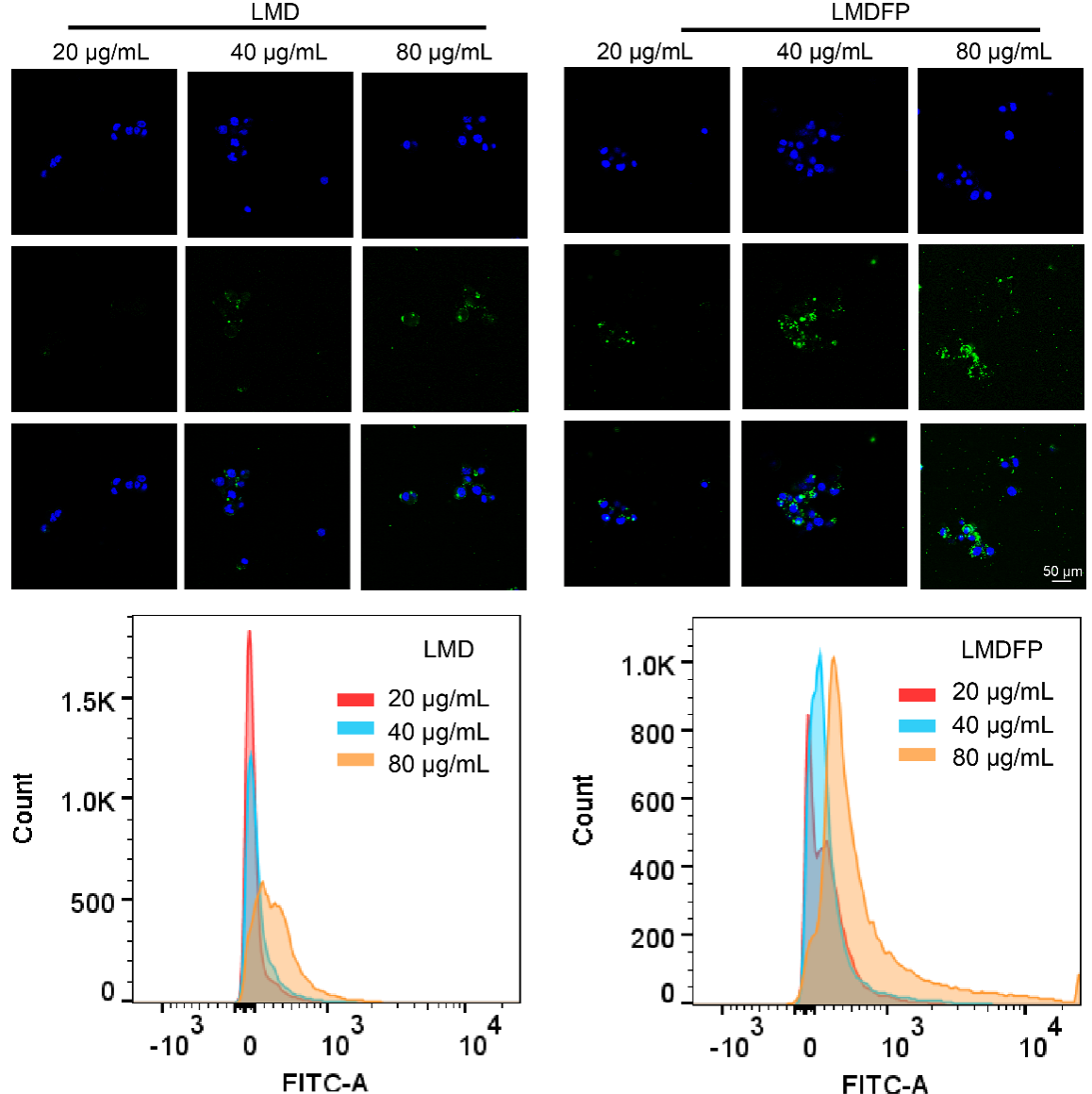


**Fig. S11.** CLSM images (blue represents nuclei while green reveals signal of FITC) and corresponding flow cytometry of cellular uptake in PC3 cells: tumor cells incubated with LMD-FITC and LMDFP-FITC at different concentrations for 4 h.


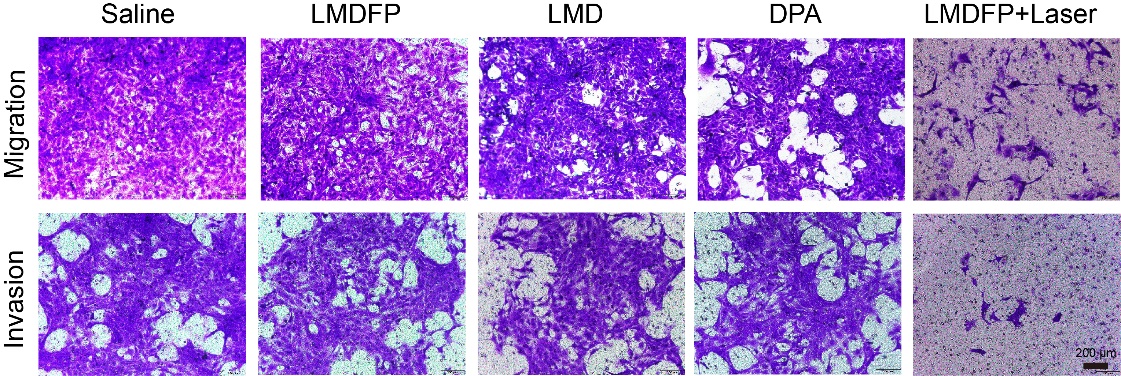


**Fig. S12.** Migration and invasion ability of 4T1 tested with transwell assay after various treatments for 48 h.


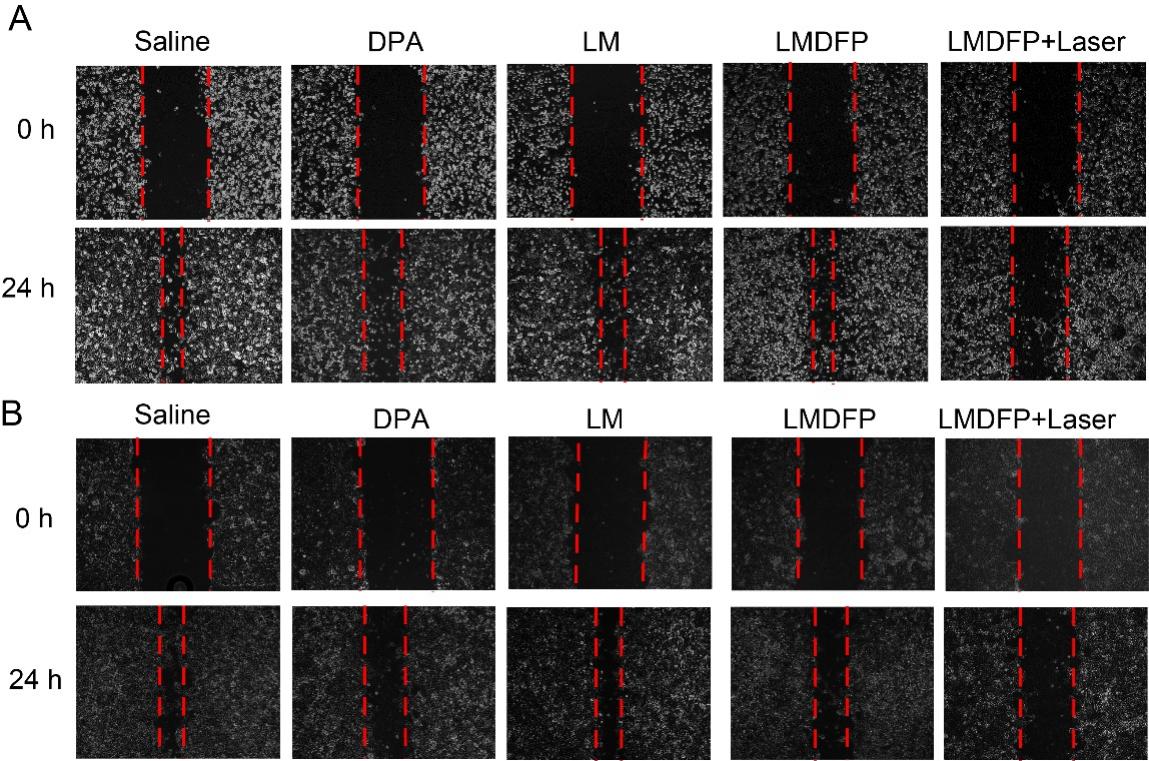


**Fig. S13.** Cell scratches assay to assess migration motility of (A) 4T1 and (B) PC3 cells with different treatments.


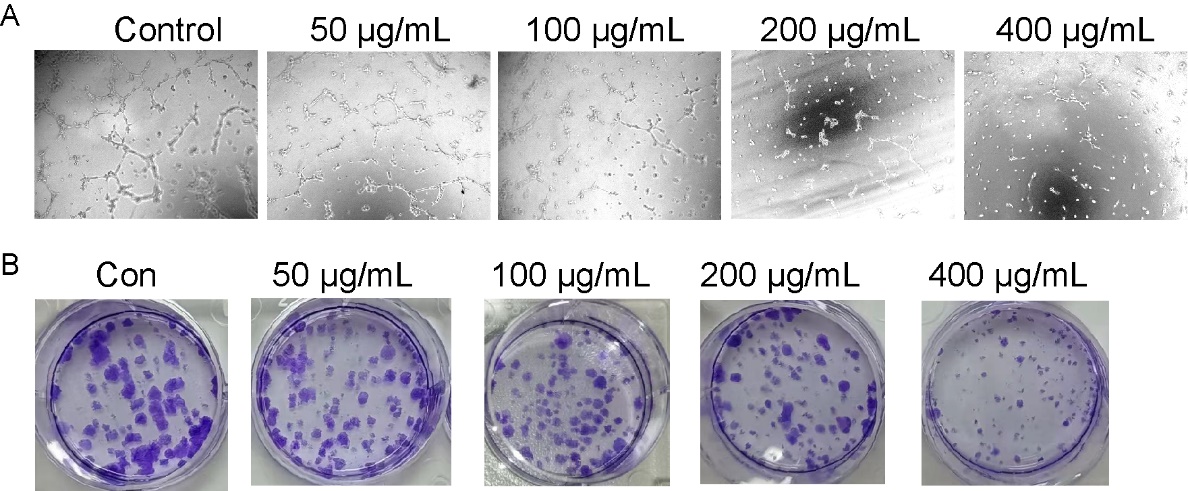


**Fig. S14.** The effect of DPA on C166 cells: Tube-formation abilities of C166 after incubated with different concentration of DPA.


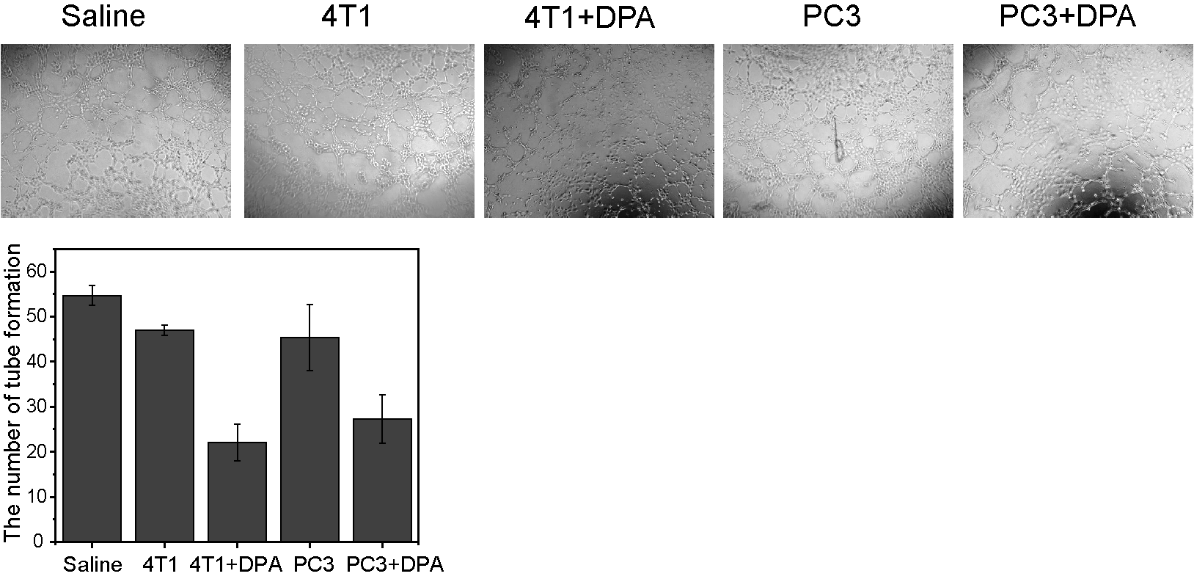


**Fig. S15.** Tube-formation abilities of C166 in different medium after cultured tumor cells.


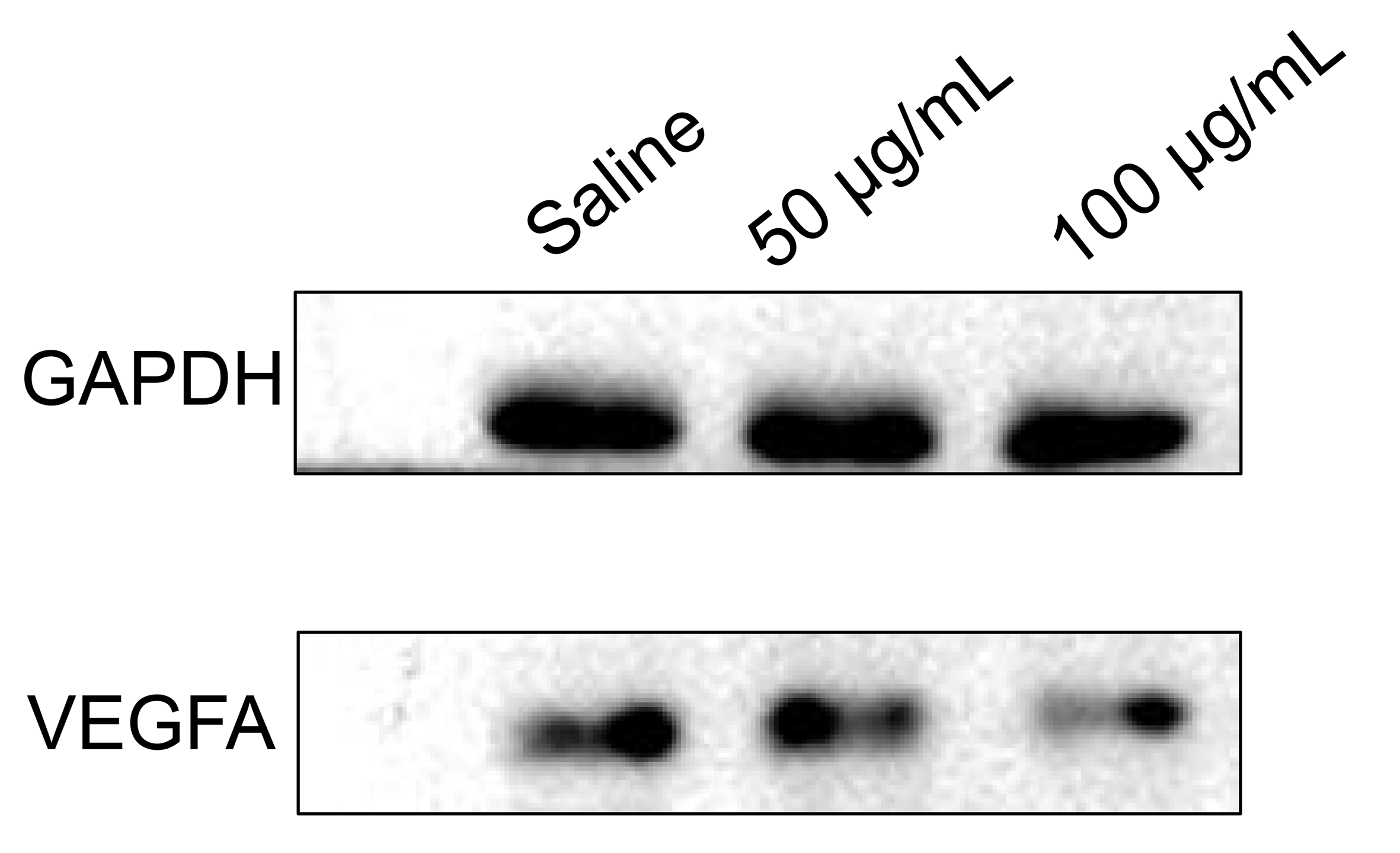


**Fig. S16.** Expression of VEGFA in 4T1 cells was analyzed by western blotting after different treatments, including saline and LMDFP at various concentrations.


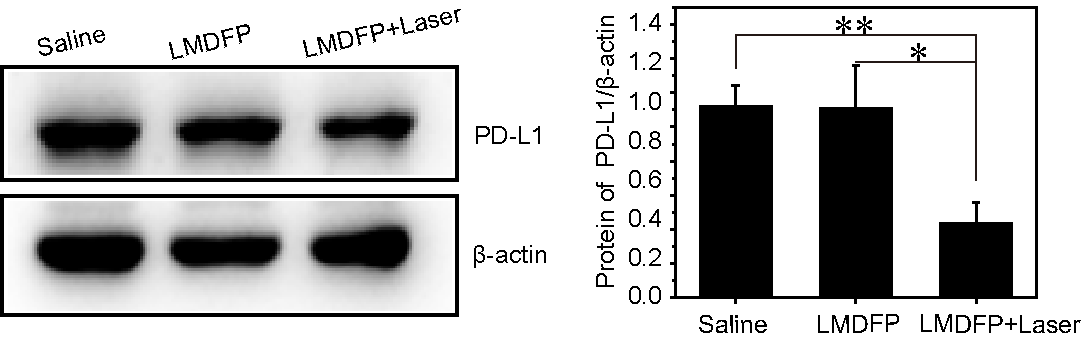


**Fig. S17.** Protein expression of PD-L1 in PC3 cells after different treatments was analyzed by western blotting and corresponding ratio of PD-L1 to β-actin was quantitatively calculated (*P < 0.05, **P <0.01).


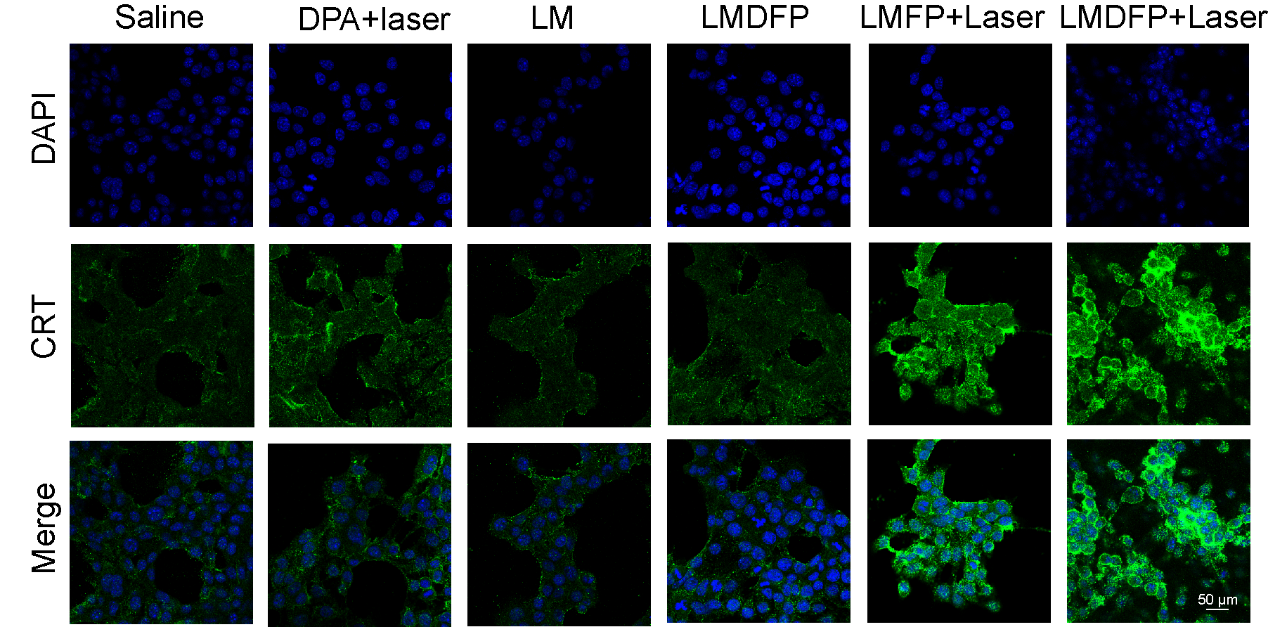


**Fig. S18.** Fluorescence stain images of CRT expression in 4T1 cells with different treatments.

.


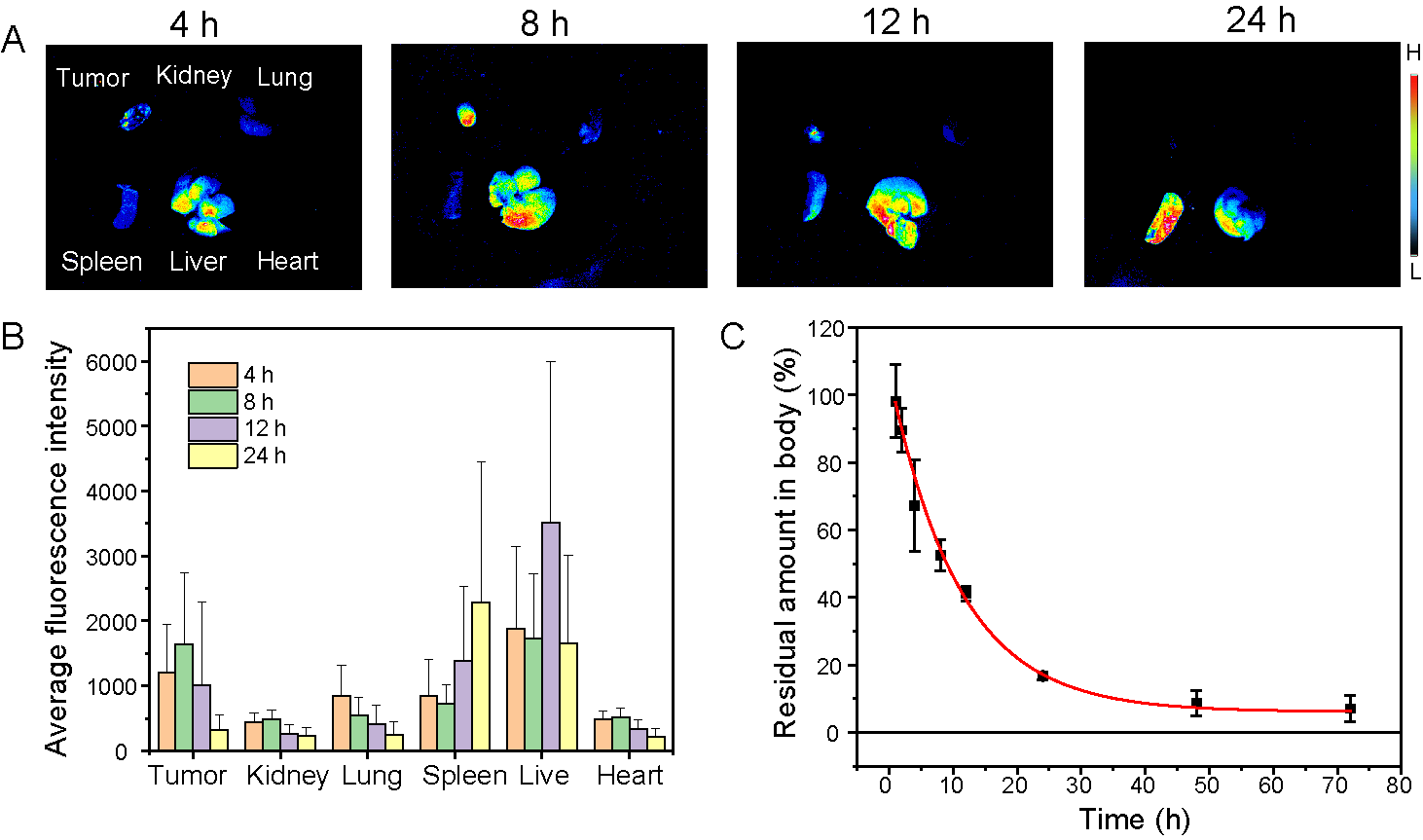


**Fig. S19.** (A, B) Ex vivo NIR-Ⅱ fluorescence imaging and corresponding fluorescence intensity of major organs and tumor dissected from mice after i.v. injected with LMDFP (10 mg/kg) for 4 h, 8 h, 12 h, 24 h, respectively. (C) Pharmacokinetics study of LMDFP in rat.


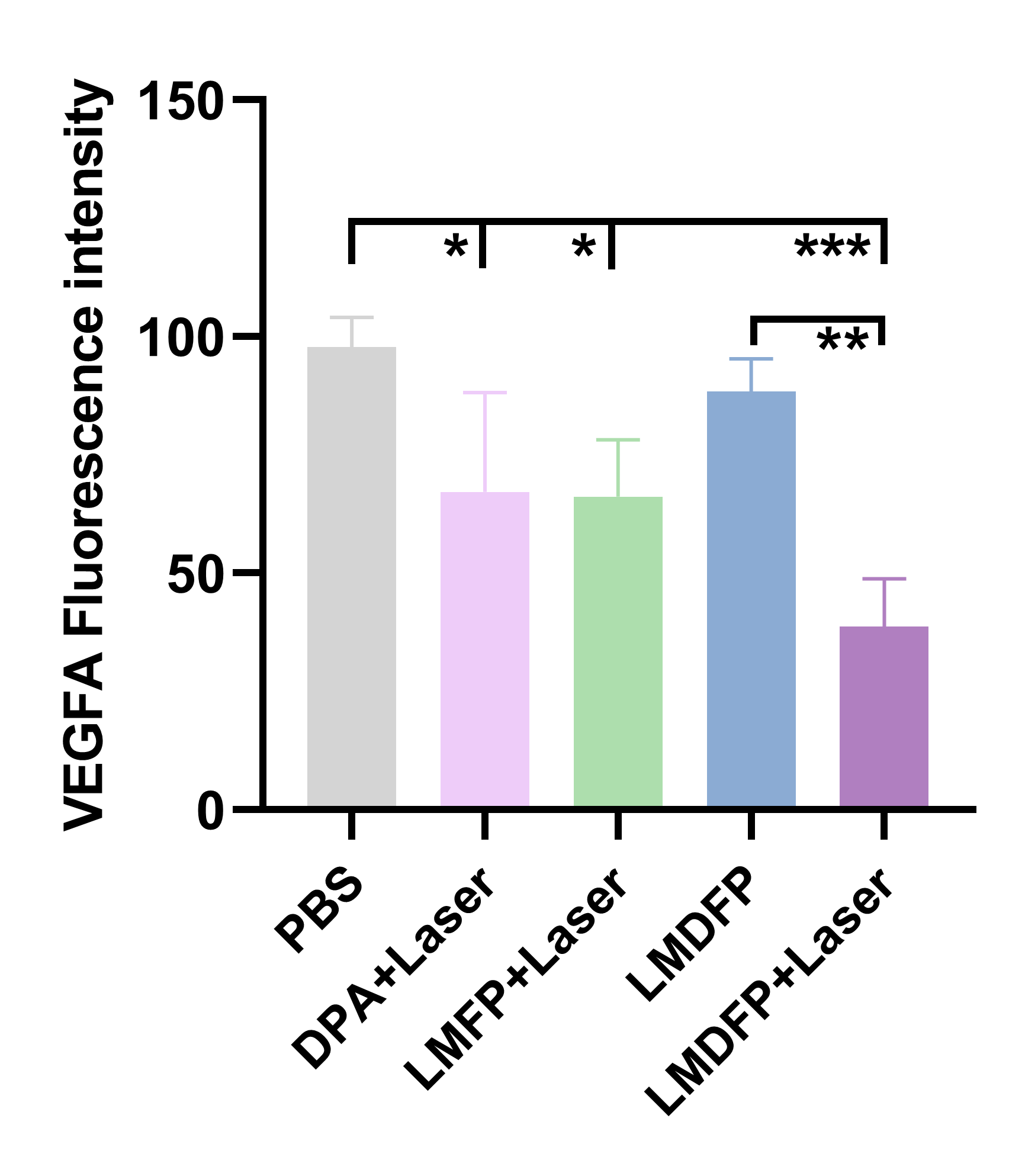


**Fig. S20.** Quantification of the inhibition of VEGFA expression in vivo calculated by ImageJ. After undergoing various treatments, the expression of VEGFA decreased significantly in the LMDFP + Laser group due to a reduction in Cu ions.

**
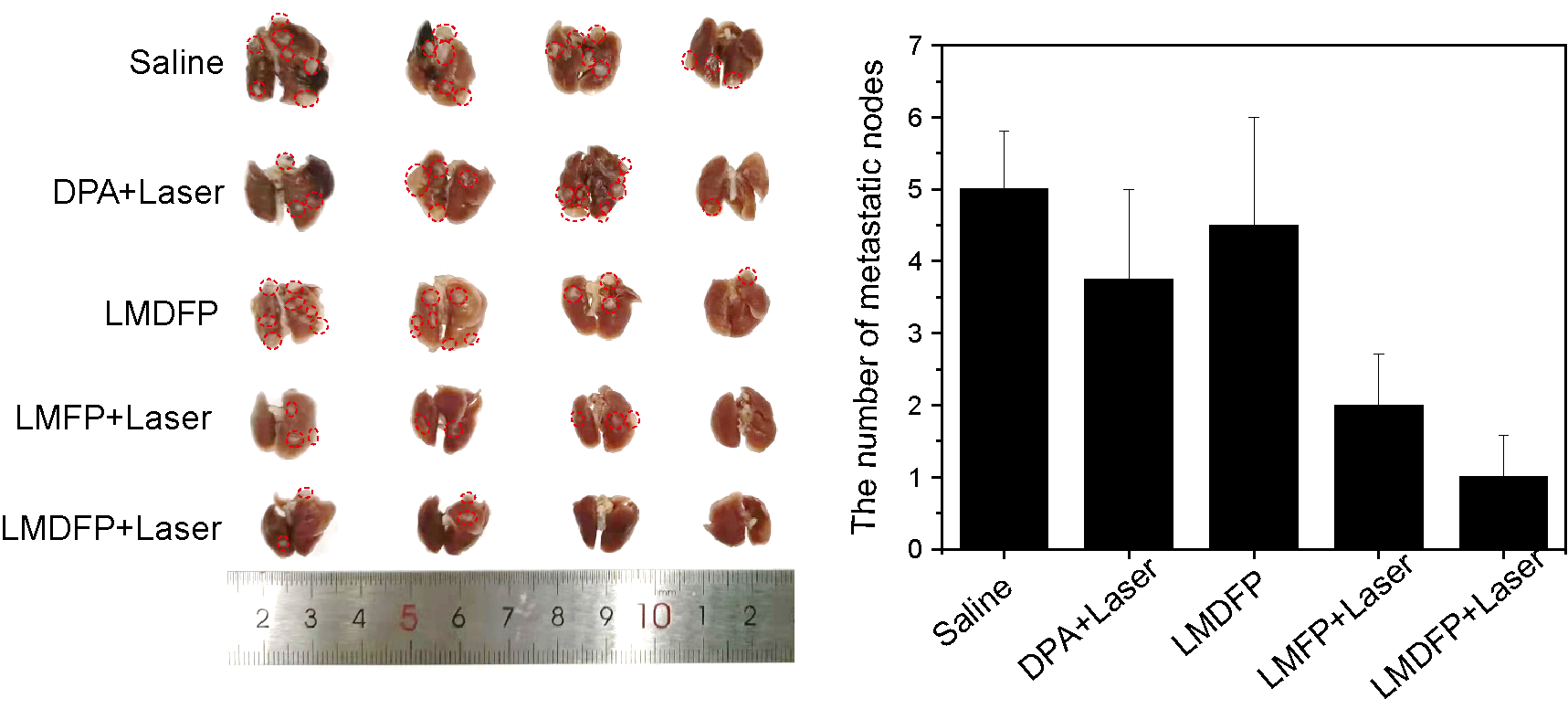
**

**Fig. S21.** Lung metastasis photograph and corresponding quantitative analysis of mouses after various treatments.


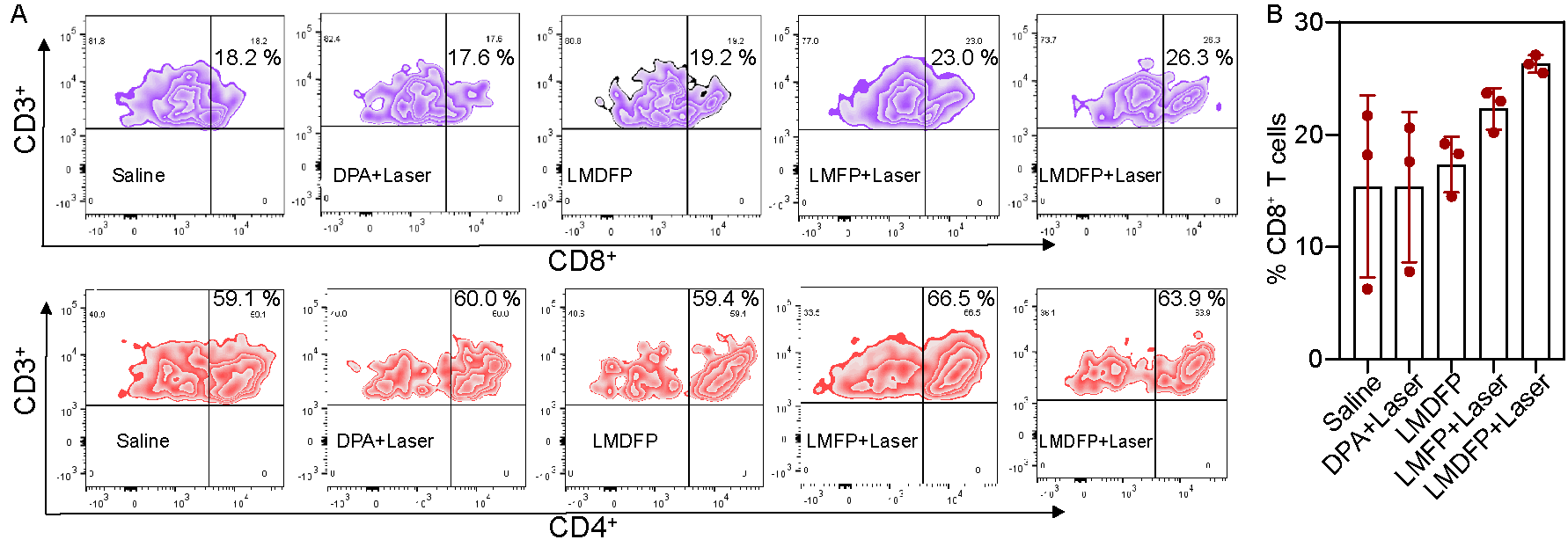


**Fig. S22.** (A) Flow cytometry data of T lymphocytes extracted from spleen tissues of 4T1 tumor loaded mouse after different treatments (first row: CD3^+^/CD8^+^; last row: CD3^+^/CD4^+^). (B) Qualification of CD3^+^/CD8^+^ cells according to (A).


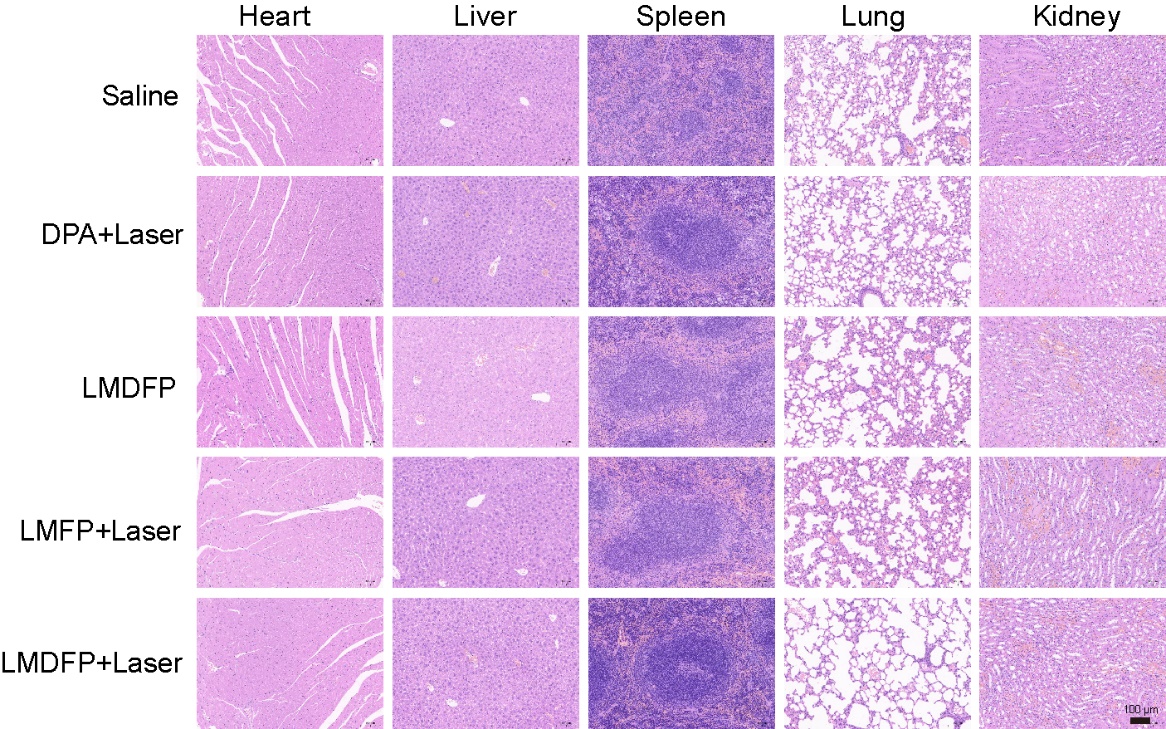


**Fig. S23.** Hematoxylin and eosin (H&E) staining pathological analysis of the vital organs including heart, liver, spleen, lung and kidney gathered from PC3 tumor-bearing nude mice after different treatments for 11 days.


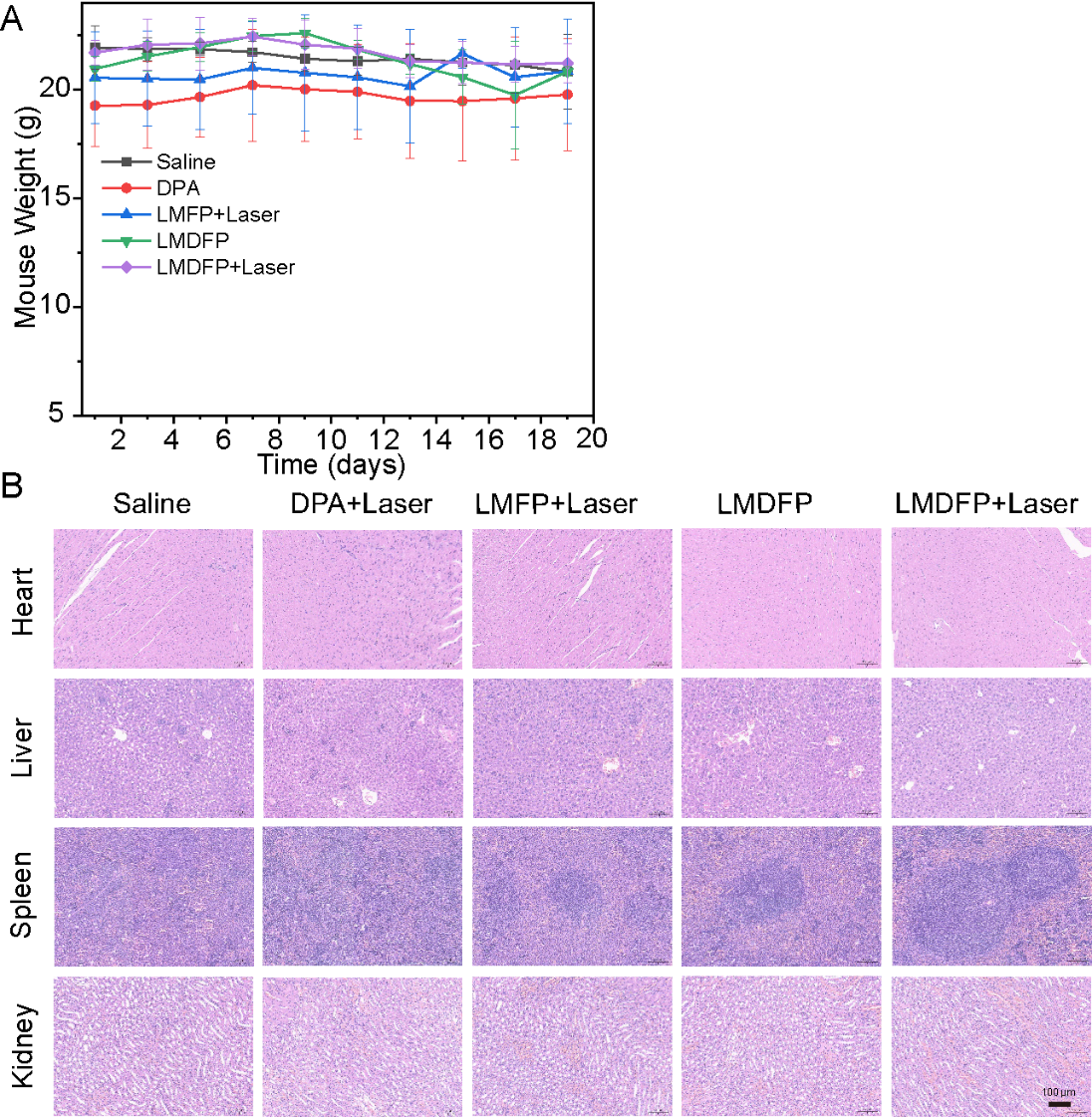


**Fig. S24.** (A) Body weight variation of Balb/C mice after various treatments during the monitoring period. (B) H&E staining pathological analysis of the vital organs including heart, liver, spleen, lung and kidney gathered from 4T1 tumor-bearing nude mice after different treatments for 19 days.


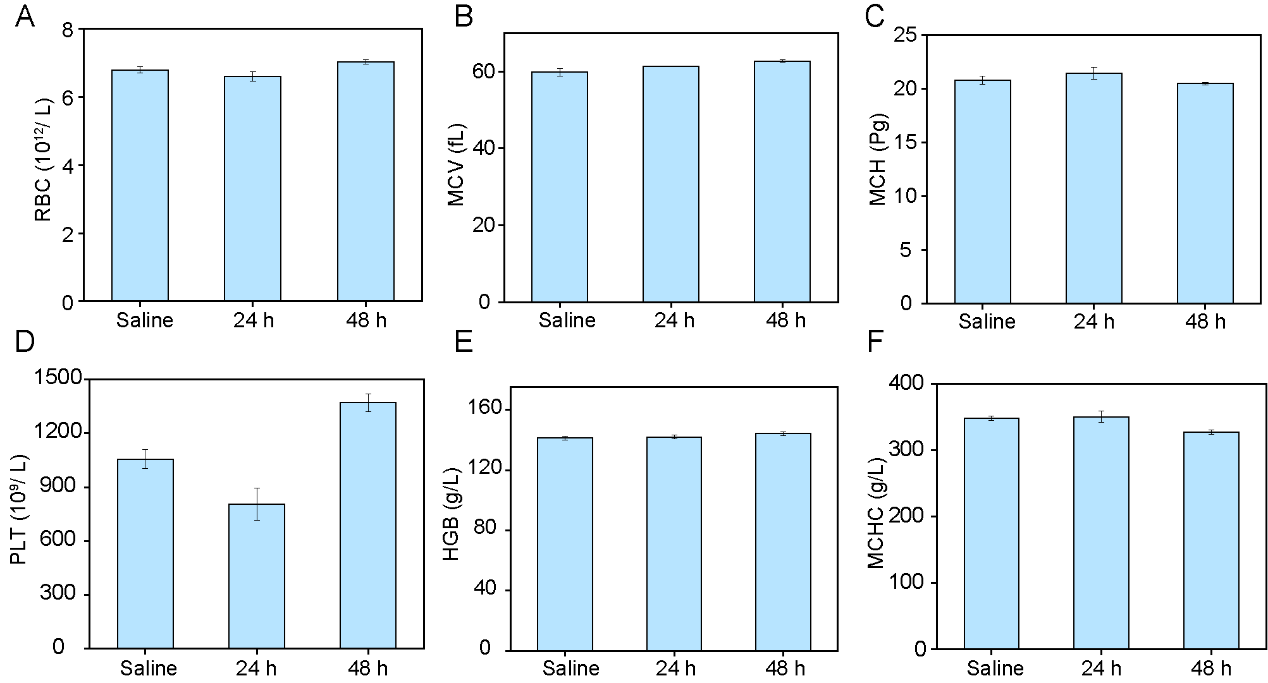


**Fig. S25.** Main blood routine indexes of mice after intravenous injection with saline or LMDFP at 24 and 48 hours.


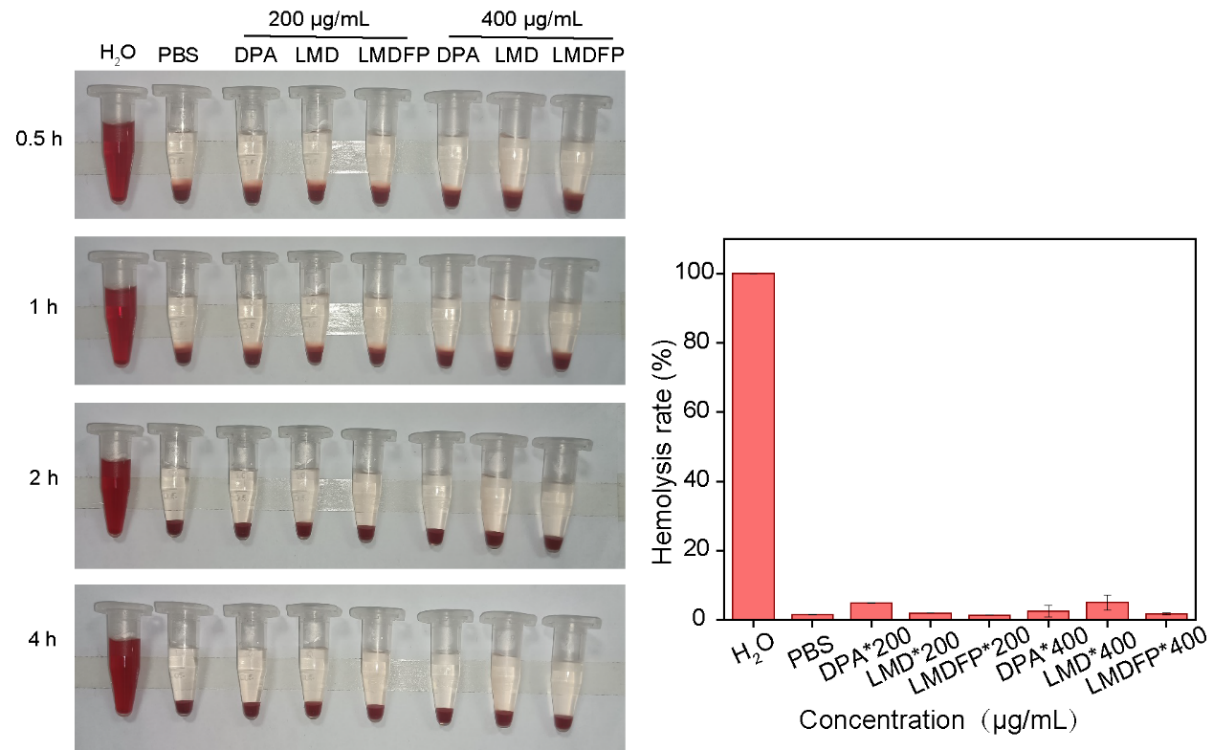


**Fig. S26.** The hemolysis assay of free DPA, LMD and LMDFP with different concentrations.
